# Recommendations from the Italian intersociety consensus on Perioperative Anesthesia Care in Thoracic surgery (PACTS) part 1: preadmission and preoperative care

**DOI:** 10.1186/s13741-020-00168-y

**Published:** 2020-12-01

**Authors:** Federico Piccioni, Andrea Droghetti, Alessandro Bertani, Cecilia Coccia, Antonio Corcione, Angelo Guido Corsico, Roberto Crisci, Carlo Curcio, Carlo Del Naja, Paolo Feltracco, Diego Fontana, Alessandro Gonfiotti, Camillo Lopez, Domenico Massullo, Mario Nosotti, Riccardo Ragazzi, Marco Rispoli, Stefano Romagnoli, Raffaele Scala, Luigia Scudeller, Marco Taurchini, Silvia Tognella, Marzia Umari, Franco Valenza, Flavia Petrini, on behalf of AIPO, Associazione Italiana Pneumologi Ospedalieri, SIAARTI, Società Italiana di Anestesia Analgesia Rianimazione e Terapia Intensiva, SIC, Società Italiana di Chirurgia, SICT, Società Italiana di Chirurgia Toracica, SIET, Società Italiana di Endoscopia Toracica, SIP, Società Italiana di Pneumologia

**Affiliations:** 1 Department of Critical and Supportive Care, Fondazione IRCCS Istituto Nazionale dei Tumori, via Venezian 1, 20133 Milan, Italy; 2 Division of Thoracic Surgery - ASST Mantova, Mantova, Italy; 3 Division of Thoracic Surgery and Lung Transplantation, Department for the Treatment and Study of Cardiothoracic Diseases and Cardiothoracic Transplantation, IRCCS ISMETT - UPMC, Palermo, Italy; 4 Department of Anesthesia and Critical Care Medicine, National Cancer Institute “Regina Elena”-IRCCS, Rome, Italy; 5 Department of Critical Care Area Monaldi Hospital, Ospedali dei Colli, Naples, Italy; 6 Division of Respiratory Diseases, IRCCS Policlinico San Matteo Foundation and Department of Internal Medicine and Therapeutics, University of Pavia, Pavia, Italy; 7 Department of Thoracic Surgery, University of L’Aquila, L’Aquila, Italy; 8 Thoracic Surgery, AORN dei Colli Vincenzo Monaldi Hospital, Naples, Italy; 9 Department of Thoracic Surgery, IRCCS Casa Sollievo della Sofferenza Hospital, San Giovanni Rotondo, FG Italy; 10 Department of Medicine, Anaesthesia and Intensive Care, University Hospital of Padova, Padua, Italy; 11 Thoracic Surgery Unit - San Giovanni Bosco Hospital - Torino, Turin, Italy; 12 Thoracic Surgery Unit, University Hospital Careggi, Florence, Italy; 13 Thoracic Surgery Unit, V Fazzi Hospital, Lecce, Italy; 14 Anesthesiology and Intensive Care Unit, Azienda Ospedaliero Universitaria S. Andrea, Rome, Italy; 15 Thoracic Surgery and Lung Transplant Unit, Fondazione IRCCS Ca’ Granda Ospedale Maggiore Policlinico, Milan, Italy; 16 Department of Morphology, Surgery and Experimental Medicine, Azienda Ospedaliero-Universitaria Sant’Anna, Ferrara, Italy; 17 Anesthesia and Intensive Care, AORN dei Colli Vincenzo Monaldi Hospital, Naples, Italy; 18 Department of Health Science, Section of Anesthesia and Critical Care, University of Florence, Florence, Italy; 19 Department of Anesthesia and Critical Care, Careggi University Hospital, Florence, Italy; 20 Pneumology and Respiratory Intensive Care Unit, San Donato Hospital, Arezzo, Italy; 21 Clinical Epidemiology Unit, Scientific Direction, Fondazione IRCCS San Matteo, Pavia, Italy; 22 Department of Thoracic Surgery, IRCCS Casa Sollievo della Sofferenza Hospital, San Giovanni Rotondo, FG Italy; 23 Respiratory Unit, Orlandi General Hospital, Bussolengo, Verona, Italy; 24 Combined Department of Emergency, Urgency and Admission, Cattinara University Hospital, Trieste, Italy; 25 Department of Critical and Supportive Care, Fondazione IRCCS Istituto Nazionale dei Tumori, Milan, Italy; 26 Department of Oncology and Onco-Hematology, University of Milan, Milan, Italy; 27 Department of Anaesthesia, Perioperative Medicine, Pain Therapy, RRS and Critical Care Area - DEA ASL2 Abruzzo, Chieti University Hospital, Chieti, Italy

**Keywords:** Perioperative care, Pneumonectomy, Practice guideline, Risk assessment, Thoracic surgery

## Abstract

**Introduction:**

Anesthetic care in patients undergoing thoracic surgery presents specific challenges that necessitate standardized, multidisciplionary, and continuously updated guidelines for perioperative care.

**Methods:**

A multidisciplinary expert group, the Perioperative Anesthesia in Thoracic Surgery (PACTS) group, comprising 24 members from 19 Italian centers, was established to develop recommendations for anesthesia practice in patients undergoing thoracic surgery (specifically lung resection for cancer). The project focused on preoperative patient assessment and preparation, intraoperative management (surgical and anesthesiologic care), and postoperative care and discharge. A series of clinical questions was developed, and PubMed and Embase literature searches were performed to inform discussions around these areas, leading to the development of 69 recommendations. The quality of evidence and strength of recommendations were graded using the United States Preventative Services Task Force criteria.

**Results:**

Recommendations for preoperative care focus on risk assessment, patient preparation (prehabilitation), and the choice of procedure (open thoracotomy vs. video-assisted thoracic surgery).

**Conclusions:**

These recommendations should help pulmonologists to improve preoperative management in thoracic surgery patients. Further refinement of the recommendations can be anticipated as the literature continues to evolve.

**Supplementary Information:**

The online version contains supplementary material available at 10.1186/s13741-020-00168-y.

## Introduction

It has become increasingly clear that thoracic surgical procedures should be considered as a single step in a long journey, the perioperative care pathway, in which the collaboration of multiple healthcare professionals is crucial. Evidence-based perioperative care protocols, known as the enhanced recovery after surgery (ERAS®) “philosophy,” have been developed in many surgical settings, including lung surgery (Batchelor et al. 2019), and shown to be effective in reducing postoperative complications and length of hospital stay (LOS) (Nicholson et al. 2014). However, systematic reviews of studies in thoracic surgery (Cerfolio et al. 2001; Das-Neves-Pereira et al. 2009; Muehling et al. 2008; Salati et al. 2012) have highlighted significant heterogeneity and methodological flaws in many trials (Fiore Jr et al. 2016; Li et al. 2017). There thus remains a need for standardized, evidence-based, pathways for perioperative care that can be updated as new evidence becomes available and new guidelines are developed and implemented.

To facilitate the development of such pathways, an Italian expert group, the Perioperative Anesthesia Care in Thoracic Surgery (PACTS) group, was convened to review the evidence supporting different preoperative, intraoperative, and postoperative interventions in thoracic surgery patients.

## Methods

The PACTS group is a joint task force of the Italian Society of Anesthesia, Analgesia, Resuscitation, and Intensive Care (Società Italiana di Anestesia Analgesia Rianimazione e Terapia Intensiva, SIAARTI); the Italian Society of Thoracic Surgery (Società Italiana di Chirurgia Toracica, SICT); the Italian Society of Thoracic Endoscopy (Società Italiana di Endoscopia Toracica, SIET); the Italian Society of Surgery (Società Italiana di Chirurgia, SIC); the Italian Association of Hospital Pneumologists (Associazione Italiana Pneuomologi Ospedalieri, AIPO); and the Italian Society of Pneumology (Società Italiana di Pneumologia, SIP/IRS). The group comprised 13 anesthetists, 3 pneumologists, 8 thoracic surgeons, a clinical epidemiologist, and management staff. Clinical centers were chosen from the participating Societies according to their expertise in thoracic surgery (primarily in cancer patients) in Italy.

A Delphi consensus method was used to achieve consensus. Two preparatory meetings, three Delphi rounds, and a final consensus conference took place between June 2018 and March 2019. In the preparatory meetings, decisions were made on the final scope and structure of the project, composition of the expert panel, methods (including relevant clinical questions framed according to the PICO [patients, intervention, comparison, outcome] framework), definitions, authorship criteria, and management of potential conflicts of interest. It was decided that the principal focus of the project would be on elective lung resection for lung cancer in adults, and that the project would address three main areas: preoperative patient assessment and preparation, intraoperative management (surgical and anesthesiologic), and postoperative procedures and discharge. Surgery for mediastinal masses, esophagectomy, diagnostic procedures, and lung transplantation were not included in the project. An experienced medical librarian performed PubMed and Embase literature searches before the Delphi rounds, and updated them regularly; additional evidence was identified by manually scanning the reference lists in retrieved papers. A shared online folder was used to make evidence available to all group members. The search strategies are available in the Additional file 1.

The panel was divided into groups, covering preadmission, preoperative, intraoperative, and postoperative care. Each group comprised anesthesiologists, thoracic surgeons, and a pulmonologist. In the preliminary Delphi round, each group was assigned a specific subgroup of questions for each area, and drafted preliminary recommendations in response to each question, supported by appropriate references. In addition, a preliminary assessment of the overall quality of the evidence for each question was made at this time. In subsequent Delphi rounds, the whole panel had the opportunity to suggest modifications to any of the questions and recommendations.

The panel adopted the United States Preventative Services Task Force (USPFTF) system for grading the quality of evidence (Table 1) and strength of recommendations (Table 2) (United States Preventative Services Task Force 2019). In addition, the panel classified as “Best Practice” the Recommendations that according to the panel presented a high level of certainty despite little or no direct evidence being available. A final consensus conference was held on 19 March 2019, in which the panel finalized the wording, quality of evidence, and strength of each recommendation. Consensuses were based on pre-specified decision rules, according to the strength of the recommendation as defined in Table 2. For grades A, B, or C recommendations, consensus required > 70% A/B/C ratings with < 15% D/I ratings. For grade D or I recommendations, consensus required > 70% D/I ratings and < 15% A/B/C ratings. The USPFTF system was used in preference to the GRADE system, which has been used in the ERAS lung surgery guidelines (Batchelor et al. 2019), because the intention was to produce a position statement rather than full practice guidelines. The GRADE system involves full appraisal of a limited number of PICO questions, and is therefore time- and resource-consuming. It is not always feasible where a number of recommendations are required in fields where no large evidence base exists, or which cannot easily be addressed using a PICO framework.

**Table 1:** Grading of quality of evidence according to United States Preventative Services Task Force (USPSTF) criteria (United States Preventative Services Task Force 2019)

Level of evidence	Definition
Good	The available evidence usually includes consistent results from well-designed, well-conducted studies in representative primary care populations. These studies assess the effects of the preventive service on health outcomes. *This conclusion is therefore unlikely to be strongly affected by the results of future studies.*
Fair	The available evidence is sufficient to determine the effects of the preventive service on health outcomes, but confidence in the estimate is constrained by such factors as: the number, size, or quality of individual studies; inconsistency of findings across individual studies; limited generalizability of findings to routine primary care practice; lack of coherence in the chain of evidence. *As more information becomes available, the magnitude or direction of the observed effect could change, and this change may be large enough to alter the conclusion.*
Poor	The available evidence is insufficient to assess effects on health outcomes. Evidence is insufficient because of: the limited number or size of studies; important flaws in study design or methods; inconsistency of findings across individual studies; gaps in the chain of evidence; findings not generalizable to routine primary care practice; lack of information on important health outcomes. *More information may allow estimation of effects on health outcomes.*

**Table 2:** Strength of recommendations (USPSTF criteria) (United States Preventative Services Task Force 2019)

Grade	Definition	Suggestion for practice
A	The USPSTF recommends the service. There is high certainty that the net benefit is substantial.	Offer or provide this service
B	The USPSTF recommends the service. There is high certainty that the net benefit is moderate or there is moderate certainty that the net benefit is moderate to substantial.	Offer or provide this service
C	The USPSTF recommends selectively offering or providing this service to individual patients based on professional judgement and patient preferences. These are at least moderate certainty that the net benefit is small.	Offer or provide this service for selected patients depending on individual circumstances
D	The USPSTF recommends against the service. There is moderate or high certainty that the service has no net benefit or that the harms outweigh the benefits.	Discourage the use of this service
I	The USPSTF concludes that the current evidence is insufficient to assess the balance of benefits and harms of the service. Evidence is lacking, of poor quality, or conflicting, and the balance of benefits and harms cannot be determined.	Read the clinical considerations section of USPSTF Recommendation Statement. If the service is offered, patients should understand the uncertainty about the balance of benefits and harms

After the consensus meeting, a draft report was prepared and distributed via email to supervisors and the steering committee for modification and comment. Based on this feedback, a second draft was prepared and distributed to the expert panel and external consultants. Each author approved the final version prior to submission. This paper summarizes the final recommendations for preadmission and preoperative care, and the supporting evidence for these (Table 3). The recommendations for intraoperative and postoperative care are presented in an accompanying paper.

**Table 3:** List of recommendations for preadmission and preoperative care

Recommendation	Level of evidence	Strength of recommendation
Preadmission		
We recommend fully evaluating patients with lung cancer who are potential candidates for curative surgical resection, regardless of age. However, age itself is a risk factor included in two mortality risk scores after thoracic surgery, and should be taken into account to estimate the perioperative risk.	Fair	A
We recommend that patients with ASA class ≥ 3 should be considered at higher risk of developing postoperative complications.	Good	A
In obese patients, we recommend specific care for airway management, with proactive strategies to reduce the risk of cardiovascular, endocrine, metabolic, and infective complications; any effort can be fruitful, including special attention to patient-related factors. Pre-operative screening of obstructive sleep apnea (OSA) by means of validated questionnaires is suggested in high-risk obese patients, with the aim of implementing strategies to reduce perioperative and postoperative complications. The perioperative team should focus on strategies to reduce the risk of complications for patients with body mass index ≥ 30 kg/m^2^.	Good	A
We recommend identifying the patients with preoperative abnormal serum creatinine and glomerular filtration rate as high-risk patients, and implementing prophylactic strategies against acute kidney injury in these patients. Hemodialysis is not an absolute contraindication to lung resection for non-small cell lung cancer. Careful monitoring of metabolic and hematologic parameters, and prompt and aggressive treatment of complications, is recommended in the perioperative period.	Poor	A
We recommend a smoking cessation period in current smokers with lung cancer who are potential candidates for curative surgical resection. An optimal interval of cessation has not been clearly identified. Nonetheless, given that smoking status is a strong predictor of postoperative lung complications, we suggest smoking cessation at least 2–3 weeks before surgery (ideally 4 weeks before).	Fair	A
Alcohol abuse in patients undergoing lung cancer surgery is associated with increased postoperative pulmonary complications and mortality, and reduced long-term survival. In alcohol abusers, we recommend cessation of alcohol consumption at least 2–3 weeks before surgery (ideally 4 weeks before).	Fair	A
We recommend a careful preoperative cardiac evaluation—including clinical scores—in order to identify potential cardiac risk factors. Recognition of these factors allows stratification of perioperative risk, optimization of medical treatment, perioperative planning and an overall reduction in morbidity.	Fair	A
We recommend measuring both ppoFEV_1_ and ppoDLCO during preoperative respiratory risk evaluation. ppoFEV_1_ and ppoDLCO levels of 40% are considered the lower limits for safe lung surgery, except in selected cases (lung volume reduction effect) where a lower threshold (ppoFEV_1_ and ppoDLCO = 30%) may be considered. Because ppoFEV_1_ and ppoDLCO are not always accurate predictors of postoperative function and outcome, we recommend the use of a larger panel of exercise tests in patients with values < 40% to evaluate risk according to guidelines for the preoperative evaluation of lung resection patients.	Fair	A
VO2max evaluation is recommended to stratify perioperative respiratory risk. Patients having a VO2max > 20 mL/kg/min are regarded as being at low risk of pulmonary complications, and are deemed fit for major surgery. It is recommended that patients having a VO_2_max < 10 mL/kg/min should be counseled about minimally invasive surgery, sublobar resections or nonoperative treatment options. Patients having a VO_2_max between 10 and 20 mL/kg/min require further multi-dimensional steps for the stratification of respiratory risk. (Lower technology tests, such as the stair-climbing test or the shuttle walk distance, may be used instead of CEPT, but the quality of evidence is lower.)	Fair	A
Arterial blood gas analysis should be performed in all patients scheduled for an elective pulmonary resection as part of the basic pulmonary function tests.	Fair	A
We recommend evaluating diabetes and assessing preoperative nutritional status (including weight loss) to estimate the surgical risk of patients undergoing thoracic surgery.	Fair (diabetes evaluation) Good (preoperative nutritional assessment)	A
Preoperative risk stratification aims at identifying high risk surgical patients (e.g., those with ASA ≥ 3, advanced cardiac disease, renal failure, VO_2_max < 10 mL/kg/min, ppoFEV_1_ or ppoDLCO < 40%, systemic disease, or other risk factors). In these patients, multidisciplinary assessment is useful to consider different treatment options and select the best therapeutic approach.	Poor	A
We recommend preoperative exercise rehabilitation in candidates for curative surgical intervention for lung cancer as it may reduce postoperative pulmonary complications. Since prehabilitation may reduce length of stay and postoperative pulmonary complications, it may be useful in COPD patients with mild to severe airway obstruction. Multimodal prehabilitation (early functional respiratory evaluation, smoking cessation, respiratory rehabilitation, nutritional status, physical exercise) is more effective than unimodal prehabilitation. It is advisable to schedule a preoperative prehabilitation program of 3 weeks.	Poor	A
Patients’ engagement has proven benefits on both clinical outcomes and healthcare sustainability. We suggest a patient Health Engagement (PHE) model to monitor patients’ engagement and psychological needs and expectations.	Fair	B
Preoperative care		
We recommend the video-assisted thoracoscopic approach for lung surgery whenever possible, because of the lower incidence of postoperative complications, shorter length of hospital stay and lower levels of postoperative pain associated with this technique.	Good	A
We do not recommend preoperative mechanical bowel preparation in patients undergoing lung surgery.	Poor	D
We recommend limiting clear fluid and solid fasting up to 2 and 6 hours, respectively, in patients undergoing lung surgery who are not at risk of delayed gastric emptying.	Good	A
We recommend preoperative carbohydrate loading with clear fluids up to 2 h prior to surgery for patients undergoing lung surgery, especially malnourished patients, in order to reduce perioperative discomfort and insulin resistance.	Poor	A
We suggest avoiding the routine use of benzodiazepines before thoracic surgery, especially in elderly people. When used in selected cases, short-acting benzodiazepines should be preferred over long-acting agents.	Fair	B

## Preadmission care

### Risk assessment

Recommendation 1: *We recommend fully evaluating patients with lung cancer who are potential candidates for curative surgical resection, regardless of age. However*, *age itself is a risk factor included in two mortality risk scores after thoracic surgery*, *and should be taken into account to estimate the perioperative risk*.

Level of evidence: Fair

Strength of recommendation: A

The prevalence of lung cancer increases with age, and it is estimated that approximately 30–35% of candidates for pulmonary resection for lung cancer are over 70 years of age (Brunelli et al. 2013). However, resection rates in elderly patients are often lower than in younger patients, largely due to the presence of comorbidities (Baldvinsson et al. 2017). Compared with younger patients, patients aged 70–75 years or older are at higher risk of complications such as prolonged intubation, pneumonia, and cardiac arrhythmias (DeLuzio et al. 2016; Matyal et al. 2010; Puri et al. 2014; Suemitsu et al. 2009; Trinh et al. 2016). There is currently no consensus as to whether older age is associated with higher mortality after thoracic surgery (Baldvinsson et al. 2017; Puri et al. 2014; Suemitsu et al. 2009; Trinh et al. 2016; Berrisford et al. 2005; Groenendijk et al. 1999), but age is included in a number of mortality risk scoring systems, such as Thoracoscore (Falcoz et al. 2007) and Eurolung (Brunelli et al. 2017). In general, advanced age per se is not considered a contraindication to curative resection in patients with lung cancer (Brunelli et al. 2013; Groenendijk et al. 1999; Falcoz et al. 2007; Brunelli et al. 2017; Matsuoka et al. 2005), and this view has been endorsed in guidelines from a number of organizations (Brunelli et al. 2013; Brunelli et al. 2009; Pallis et al. 2010).

Recommendation 2: *We recommend that patients with ASA class* ≥ *3 should be considered at higher risk of developing postoperative complications*.

Level of evidence: Good

Strength of recommendation: A

Multiple studies have shown that an American Society of Anesthesiologists (ASA) class > 2 is a significant risk factor for death, prolonged hospitalization, or major cardiopulmonary complications in patients undergoing lung resection (DeLuzio et al. 2016; Berrisford et al. 2005; Groenendijk et al. 1999; Marret et al. 2010; Smetana 1999). A recent study using data from a large US database showed that ASA class 3 and ASA class 4–5 were both significant predictors of prolonged LOS in patients undergoing lung resection (class 3: odds ratio [OR] 1.6 [95% confidence interval [CI] 1.1–2.3], *P* = 0.007; class 4–5: OR 2.2 [95% CI 1.4–3.3] *P* = 0.001) (DeLuzio et al. 2016).

Recommendation 3: *In obese patients*, *we recommend specific care for airway management*, *and proactive strategies to reduce the risk of cardiovascular*, *endocrine*, *metabolic*, *and infective complications*; *any effort can be fruitful*, *including special attention to patient*-*related factors*. *Pre*-*operative screening of obstructive sleep apnea* (*OSA*) *by means of validated questionnaires is suggested in high*-*risk obese patients*, *with the aim of implementing strategies to reduce perioperative and postoperative complications*. *The perioperative team should focus on strategies to reduce the risk of complications for patients with body mass index* ≥ *30 kg/m*^2^*.*

Level of evidence: Good

Strength of recommendation: A

Obesity is a growing problem in developed countries, and the perioperative respiratory management of obese surgical patients poses a number of challenges.

The available evidence suggests that obesity is not associated with increased mortality and morbidity after lung resection for lung cancer, although operative times are generally longer in obese patients than in normal-weight patients (Petrini et al. 2016; Dhakal et al. 2013; Mungo et al. 2015; Paul et al. 2015; Wang et al. 2018). Indeed, data from the American College of Surgeons National Surgical Quality Improvement Program (NSQIP) database suggest that being overweight (body mass index 25–30 kg/m^2^) is actually associated with a decreased risk of prolonged LOS following lung resection, compared with being within normal weight ranges (Mungo et al. 2015), and other studies have reported greater morbidity and longer LOS in underweight patients, compared with obese or overweight patients (Mungo et al. 2015; Wang et al. 2018). Obesity per se should not, therefore, be considered a contraindication to lung resection or lobectomy (Paul et al. 2015; Wang et al. 2018). However, periprocedural assessment and critical care strategies designed specifically for obese patients are crucial for reducing perioperative morbidity and mortality (Petrini et al. 2016).

Evidence-based recommendations for the perioperative management of obese patients have been published by the SIAARTI Airway Management Study Group (Petrini et al. 2016). These emphasize the key role of the anesthetist, in cooperation with the pulmonologist, in perioperative risk management, and the importance of identifying obstructive sleep apnea (OSA) and other comorbidities that may complicate anesthesia and surgery in obese patients. In particular, mask ventilation and laryngoscopy may be difficult in obese patients, and hence a robust airway management strategy is recommended (Petrini et al. 2016). The STOP-BANG questionnaire should be used to identify patients with undiagnosed OSA, who may be at increased risk of postoperative oxygen desaturation, respiratory failure, and unplanned admission to the intensive care unit (ICU) (Petrini et al. 2016).

Recommendation 4: *We recommend identifying the patients with preoperative abnormal serum creatinine and glomerular filtration rate as high*-*risk patients*, *and implementing prophylactic strategies against acute kidney injury in these patients*. *Hemodialysis is not an absolute contraindication to lung resection*, *even if morbidity and mortality are likely to be higher*. *Careful monitoring of metabolic and hematologic parameters*, *and prompt and aggressive treatment of complications*, *is recommended in the perioperative period*.

Level of evidence: Poor

Strength of recommendation: A

Postoperative acute kidney injury (AKI) occurs in approximately 5–10% of thoracic surgery patients, and is associated with prolonged hospitalization and increased rates of complications such as tracheal reintubation, postoperative mechanical ventilation, and ICU admission; it has also been associated with increased mortality (Ahn et al. 2016; Cardinale et al. 2018; Ishikawa et al. 2012; Licker et al. 2011; Romagnoli and Ricci 2015). Baseline renal dysfunction is a critical factor associated with postoperative AKI, as it reflects the kidney’s vulnerability to injuries resulting from a diminished renal functional reserve. Several studies have investigated the prognostic impact of preoperative renal dysfunction, defined as doubling of plasma creatinine, decreased estimated glomerular filtration rate (eGFR), or requirement for renal replacement therapy (Ahn et al. 2016; Cardinale et al. 2018; Ishikawa et al. 2012; Licker et al. 2011). In a recent study, preoperative serum creatinine > 1.2 mg/dL or eGRF < 60 mL/min were found to be independent predictors of postoperative AKI (Ahn et al. 2016). According to the Kidney Disease Improving Global Outcomes (KDIGO) organization, prophylactic strategies against acute kidney injury include maintenance of volume status and adequate perfusion pressure (mean arterial blood pressure ≥ 65 mmHg); consideration of functional hemodynamic monitoring and protocol-driven management aimed at avoiding hypotension, optimization of cardiac output, and oxygen delivery; maintenance of normal serum lactate levels (< 2 mmol/L); avoidance of hyperglycemia (< 150 mg/dl); monitoring serum creatinine and urine output; and avoidance or discontinuation of nephrotoxic agents whenever possible (Kidney Disease Improving Global Outcomes (KDIGO) 2012).

Anemia is common in patients with chronic kidney disease, especially in advanced stages. Iron deficiency is frequent in such patients and should be corrected before surgery, principally with iron therapy. Erythropoiesis-stimulating agents may be useful to treat anemia on an individualized basis, avoiding hemoglobin levels below 9.0–10.0 g/dL (Kidney Disease Improving Global Outcomes (KDIGO) Anemia Workgroup 2012).

Evidence from small patient series shows that pulmonary resection for non-small cell lung cancer in patients receiving hemodialysis is associated with high rates of morbidity and mortality (75% and 10%, respectively) (Akiba et al. 2010; Ciriaco et al. 2005; Matsuoka et al. 2013a; Park et al. 2015). Although radical lung resection appears to be safe in selected patients, careful metabolic, hematologic, and pharmacological management is mandatory during the perioperative period.

Recommendation 5: *We recommend a smoking cessation period in current smokers with lung cancer who are potential candidates for curative surgical resection*. *An optimal interval of cessation has not been clearly identified. Nonetheless*, *given that smoking status is a strong predictor of postoperative lung complications*, *we suggest smoking cessation at least 2*-*3 weeks before surgery* (*ideally 4 weeks before*).

Level of evidence: Fair

Strength of recommendation: A

Current smoking is a risk factor for postoperative complications, prolonged LOS, and mortality in patients undergoing thoracic surgery (Brunelli et al. 2013; Agostini et al. 2018). Several studies have shown that active smoking increases the risk of postoperative complications such as pneumonia, failure to wean from ventilator, or reintubation after lung surgery (Barrera et al. 2005; Eichenbaum and Neustein 2010; Gajdos et al. 2012; Harpole Jr et al. 1999). In a study of 300 cancer patients undergoing lung resection, such complications occurred in 23% of current smokers, compared with 8% of nonsmokers and 19% of ex-smokers who had stopped smoking more than 2 months before surgery (Barrera et al. 2005). However, one prospective observational study found no difference between current or past smokers in terms of postoperative complication rates and pulmonary function 1 year after lung resection for cancer (Groth et al. 2009).

Smoking abstinence for at least 4 weeks may be associated with reduced perioperative respiratory complications (Brunelli et al. 2013). However, the optimal timing for smoking cessation before surgery is not clear. Berrera et al. found that smoking cessation between 1 week and 2 months before surgery reduces postoperative pneumonia (Barrera et al. 2005), and a registry-based study involving approximately 8000 patients found a trend toward decreased perioperative mortality with increasing duration of smoking abstinence prior to surgery (Mason et al. 2009).

Unfortunately, some patients do not stop smoking before surgery, despite participating in multidisciplinary smoking cessation programs. In such cases, smoking should not be considered an absolute contraindication to lung surgery.

Recommendation 6: *Alcohol abuse in patients undergoing lung cancer surgery is associated with increased postoperative pulmonary complications and mortality*, *and reduced long*-*term survival*. *In alcohol abusers*, *we recommend cessation of alcohol consumption at least 2*- *weeks before surgery* (*ideally 4 weeks before*).

Level of evidence: Fair

Strength of recommendation: A

Several studies have shown that alcohol abuse in patients undergoing surgery for lung cancer is associated with increased rates of postoperative pulmonary complications and reduced long-term survival (Batchelor et al. 2019). There is limited evidence that intensive interventions aimed at complete abstinence from alcohol for at least 4 weeks before surgery reduce postoperative complication rates, but have little effect on mortality or LOS (Egholm et al. 2018). However, the optimal timing of such interventions remains to be determined (Batchelor et al. 2019).

Recommendation 7: *We recommend a careful preoperative cardiac evaluation*—*including clinical scores*—*in order to identify potential cardiac risk factors*. *Recognition of these factors allows stratification of perioperative risk*, *optimization of medical treatment*, *perioperative planning*, *and an overall reduction in morbidity*.

Level of evidence: Fair

Strength of recommendation: A

Major cardiac adverse events, particularly supraventricular arrhythmias, are among the most common complications in patients undergoing thoracic surgery (Cagirici et al. 2005). Such events may occur after both lung resection (Kitamura et al. 2017) and less invasive procedures such as video-assisted thorascopic lobectomy (Sandri et al. 2017). Atrial fibrillation following pulmonary surgery can lead to hemodynamic instability and longer ICU and hospital stay (Frendl et al. 2014). Furthermore, cardiac disease or atrial fibrillation are common comorbidities in patients undergoing thoracic surgery, and are major risk factors for postoperative morbidity and mortality (Kristensen et al. 2014). However, the literature on cardiac risk and lung cancer surgery is scarce, particularly with respect to the impact of cardiac comorbidities on outcomes.

Preoperative cardiovascular evaluation is essential to identify patients at high risk of cardiac complications following thoracic surgery, who require careful risk stratification and optimal management based on published guidelines (Frendl et al. 2014; Kristensen et al. 2014; De Hert et al. 2018; Duceppe et al. 2017; Fleisher et al. 2014). A number of cardiac risk scores may be used for risk stratification, including the Revised Cardiac Risk Index (RCRI) (Brunelli et al. 2013), the Thoracic RCRI (ThRCRI) (Brunelli et al. 2010), and the NSQIP Index (Kristensen et al. 2014; De Hert et al. 2018; Duceppe et al. 2017). In patients with reduced functional capacity, the cardiovascular evaluation could be based on VO_2_max during cardiopulmonary exercise testing (CPET); patients with VO_2_max > 20 mL/kg/min, 10–15 mL/kg/min, and < 10 mL/kg/min may be considered as being at low, intermediate, and high risk, respectively (Spyratos et al. 2014). Simpler tests, such as the stair-climbing test or the shuttle walk distance, may also be used, but the strength of evidence supporting these tests is lower than for CPET (Kristensen et al. 2014; Spyratos et al. 2014).

Recommendation 8: *We recommend measuring both ppoFEV*_*1*_ *and ppoDLCO during preoperative respiratory risk evaluation. ppoFEV*_1_ *and ppoDLCO levels of 40*% *are considered the lower limits for safe lung surgery*, *except in selected cases* (*lung volume reduction effect*) *where a lower threshold* (*ppoFEV*_1_ *and ppoDLCO* = *30*%) *may be considered*. *Because ppoFEV*_1_ *and ppoDLCO are not always accurate predictors of postoperative function and outcome*, *we recommend the use of a larger panel of exercise tests in patients with values < 40*% *to evaluate risk according to guidelines for the preoperative evaluation of lung resection patients*.

Level of evidence: Fair

Strength of recommendation: A

Spirometric measurement of forced expiratory volume in 1 second (FEV_1_) and predicted postoperative FEV_1_ (ppoFEV_1_) has traditionally been a key component of the preoperative evaluation of lung cancer patients, and decreases in these measures are associated with increased morbidity and mortality (Brunelli et al. 2013). Similarly, carbon monoxide diffusing capacity (DLCO) and ppoDLCO are predictive of pulmonary complications after lung resection in patients without chronic obstructive pulmonary disease (COPD) (Ferguson et al. 2009; Ferguson and Vigneswaran 2008). However, these measures are not always accurate predictors of postoperative function, morbidity, and mortality. Studies have found that ppoFEV_1_ and ppoDLCO are poor predictors of postoperative lung function in patients undergoing pneumonectomy (Brunelli et al. 2007a), and that preoperative FEV_1_ and DLCO are significant predictors of pulmonary complications following lobectomy performed via thoracotomy but not thoracoscopy (Berry et al. 2010). In general, ppoDLCO appears to be the best predictor of postoperative morbidity and mortality, both in patients with or without COPD (Brunelli et al. 2013; Ferguson et al. 2009; Ferguson and Vigneswaran 2008). It is noteworthy that, in COPD patients showing a heterogeneous emphysema phenotype, ppoFEV_1_ underestimates postoperative lung function, possibly due to the so-called lobar volume reduction effect (Brunelli et al. 2013; Brunelli et al. 2009).

There is currently no consensus about FEV_1_ and DLCO thresholds that are predictive of respiratory complications. In the absence of other comorbidities, patients with FEV_1_ and DLCO values > 80% can be regarded as being at low risk in all types of thoracic surgery. Importantly, however, DLCO is a significant predictor of pulmonary complications even in patients with normal FEV_1_: more than 40% of patients with FEV_1_ > 80% have a DLCO < 80%, and approximately 7% may have a DLCO below 40% (Brunelli et al. 2013; Brunelli et al. 2009). Some authors have suggested that patients with ppoFEV_1_ and ppoDLCO > 60% may be regarded as low-risk patients (Brunelli et al. 2013; Salati and Brunelli 2016). Conversely, patients with ppoFEV_1_and ppoDLCO < 40% predicted are usually considered to be at high risk of postoperative morbidity and mortality (Beccaria et al. 2001; Fujiu et al. 2003; Wyser et al. 1999). Some authors have suggested that, depending on the expertise and facilities available, this threshold can be lowered to 30% predicted in selected patients without COPD in whom lung volume reduction is planned (Brunelli et al. 2013; Brunelli et al. 2009). For these reasons, we recommend that patients with reduced pulmonary function should undergo cardiopulmonary testing, or lower technology tests. The preoperative risk evaluation should include an assessment of COPD and its severity, where present.

In patients undergoing lobectomy, postoperative FEV_1_ and DLCO can be predicted using simple formulae that take into account the number of functional or unobstructed lung segments (Brunelli et al. 2013; Brunelli et al. 2009; Sekine et al. 2003). However, this approach overestimates the decrease in lung function following bi-lobectomy or pneumonectomy, and in these situations lung perfusion scintigraphy is recommended to estimate preoperative lung perfusion and postoperative pulmonary function (Brunelli et al. 2013; Brunelli et al. 2009; van Tilburg et al. 2009).

Recommendation 9: *VO*_2_*max evaluation is recommended to stratify perioperative respiratory risk. Patients having a VO*_2_*max* > *20 mL*/*kg*/*min are regarded as being at low risk of pulmonary complications, and are deemed fit for major surgery*. *It is recommended that patients having a VO*_2_*max* < *10 mL*/*kg*/*min should be counseled about minimally invasive surgery*, *sublobar resections*, *or nonoperative treatment options*. *Patients having a VO*_*2*_*max between 10 and 20 mL*/*kg*/*min require further multi-dimensional steps for the stratification of respiratory risk.* (*Lower technology tests*, *such as the stair*-*climbing test or the shuttle walk distance*, *may be used instead of CEPT*, *but the quality of evidence is lower*.)

Level of evidence: Fair

Strength of recommendation: A

CPET is recommended in current guidelines for the preoperative evaluation of patients with compromised pulmonary function (Brunelli et al. 2013; Brunelli et al. 2009). Patients with a VO_2_max > 20 mL/kg/min are at low risk of postoperative pulmonary complications, and these patients are deemed to be suitable candidates for all types of resection, including pneumonectomy (Brunelli et al. 2013; Brunelli et al. 2009; Salati and Brunelli 2016; Wyser et al. 1999; Torchio et al. 1998). By contrast, patients with VO_2_max < 10 mL/kg/min are at high risk of pulmonary complications, and major anatomical resection is contraindicated in these patients (Brunelli et al. 2013; Brunelli et al. 2009; Wyser et al. 1999; Puente-Maestu et al. 2011). It is recommended that these patients should be counseled about minimally invasive surgery, sublobar resections, or nonoperative treatment options for their lung cancer (Brunelli et al. 2013). Patients with VO_2_max values between 10 and 20 mL/kg/min require further risk stratification using the slope of the minute ventilation to carbon dioxide output ratio (VE/VCO_2_: also known as the ventilatory efficiency curve); a VE/VCO_2_ ratio > 35% indicates an intermediate or high risk of pulmonary complications (Salati and Brunelli 2016).

Although CPET is considered the gold standard for preoperative evaluation of lung surgery patients (Salati and Brunelli 2016), it requires specialized facilities that may not be available in all centers. Hence, several groups have used simpler tests, such as the stair-climbing test, the shuttle walk distance, or the 6-min walking test for preoperative evaluations. However, there is no consensus regarding the performance levels on these tests that would indicate a low risk of postoperative complications. The available evidence suggests that patients who are able to climb 12–14 m (approximately three flights of stairs) are at low risk of complications following pneumonectomy or lobectomy, whereas an inability to climb one flight of stairs corresponds to a VO_2_max of < 10 mL/kg/min, and hence represents a contraindication to major resection (Brunelli et al. 2013; Brunelli et al. 2007b). Similarly, a distance of 25 shuttles or > 400 m on the shuttle walk test indicates a low risk of postoperative complications (Brunelli et al. 2013). Studies evaluating the 6-mi walking test in lung resection candidates have reported conflicting results (Brunelli et al. 2013; Hattori et al. 2017).

Patients with lung cancer and a VO_2_max < 10 mL/kg/min who are being considered for curative surgery may be candidates for minimally invasive surgery or sublobar anatomic resections (segmentectomies), rather than major pneumonectomy (Brunelli et al. 2013). Both of these have been shown to reduce the risk of perioperative complications, compared with open thoracotomy, and to provide oncological outcomes that are at least comparable with those achievable with major resection (Brunelli et al. 2013). In a retrospective review of approximately 13,000 patients in the UK, patients undergoing lobectomy by video-assisted thoracic surgery (VATS) had significantly lower complication rates than those undergoing thoracotomy; similarly, other studies have shown that thoracoscopic resection or anatomical segmentectomy are associated with lower rates of mortality and pneumonia, and shorter ICU or hospital stays, compared with open thoracotomy (Brunelli et al. 2013; Oparka et al. 2013). However, limited resection carries a higher rate of loco-regional recurrences than lobectomy or pneumonectomy: recurrence rates of up to approximately 30% have been reported in patients undergoing segmentectomy, compared with < 20% in lobectomy patients (De Zoysa et al. 2012). In elderly lung cancer patients, limited resection or VATS techniques provide an alternative to standard thoracotomy, and offer the potential for quicker recovery with comparable morbidity and mortality rates (De Zoysa et al. 2012; Jaklitsch et al. 2004). An analysis from the US Surveillance, Epidemiology, and End Results (SEER) database showed that, in elderly patients with stage T1a non-small cell lung cancer, 5-year cancer-specific survival rates in patients undergoing wedge resection were comparable to those achieved in patients undergoing segmentectomy (hazard ratio [HR] 1.01; 95% CI 0.62–1.63; *P* = 0.972) or lobectomy (HR 0.98; 95% CI 0.69–1.39; *P* = 0.908) (Razi et al. 2016).

Recommendation 10: *Arterial blood gas analysis should be performed in all patients scheduled for an elective pulmonary resection as part of the basic pulmonary function tests.*

Level of evidence: Fair

Strength of recommendation: A

Arterial blood gas analysis is an objective test for the evaluation of respiratory function, and is easy to perform during the perioperative period. It is used to provide a preoperative reference for early postoperative phase management. Measurement of partial pressure of arterial oxygen (PaO_2_) and carbon dioxide (PaCO_2_) should be performed as part of the preoperative evaluation in all patients scheduled for elective pulmonary resection (Della Rocca et al. 2016; Licker et al. 2014). However, there is no consensus regarding the cut-off value of arterial oxygen tension that clearly indicates an increased risk for pulmonary resection. Traditionally, a PaO_2_ < 60 mmHg, or a PaCO_2_ > 45–50 mmHg have been considered thresholds for pulmonary resection (Della Rocca et al. 2016; Slinger and Johnston 2001). However, although a PaCO_2_ > 45 mmHg is associated with an increased risk of postoperative complications, it is not considered a contraindication to lung resection surgery (Della Rocca et al. 2016).

Recommendation 11: *We recommend evaluating diabetes and assessing preoperative nutritional status* (*including weight loss*) *to estimate the surgical risk of patients undergoing thoracic surgery*.

Level of evidence: Fair (diabetes evaluation); Good (preoperative nutritional assessment)

Strength of recommendation: A

Preoperative assessment of diabetic status is important for all surgical patients. In a retrospective review of 957 patients undergoing surgery for lung cancer, diabetes was present in 13% of patients, and was associated with significantly higher 30-day mortality, compared with patients without diabetes (7.4% vs. 3.2%, respectively, *P* = 0.04) (Washington et al. 2013). However, diabetes had no significant effect on 5-year mortality, overall mortality, or loco-regional recurrence rates. In a further study, involving approximately 8000 patients included in the NSQIP database, type 1 diabetes was found to be a significant predictor of prolonged LOS on univariate analysis (OR 1.54, *P* = 0.023), but not on multivariate analysis (DeLuzio et al. 2016).

Several studies have shown that preoperative weight loss and malnutrition status are independent risk factors for postoperative complications after thoracic surgery (Harpole Jr et al. 1999; Jagoe et al. 2001; Matsuoka et al. 2013b; Ramos et al. 2018; Watanabe et al. 2018). In a recent study involving 219 patients who had undergone major resection for lung cancer, patients with low scores on the Nutritional Risk Index (NRI) had significantly higher rates of postoperative complications (particularly pneumonia), longer chest drainage time, and longer LOS than non-malnourished patients (Ramos et al. 2018). A further study, involving 131 elderly (≥ 75 years) patients undergoing surgery for lung cancer, found significantly shorter 5-year cancer-specific survival in malnourished patients, compared with non-malnourished patients (47.8% vs. 76.2%, respectively, *P* = 0.017) (Watanabe et al. 2018). Poor nutritional status has also been reported to be a risk factor for prolonged hospitalization in patients undergoing VATS for secondary pneumothorax (Matsuoka et al. 2013b).

Recommendation 12: *Preoperative risk stratification aims at identifying high risk surgical patients* (*e*.*g*. *those with ASA* ≥ *3, advanced cardiac disease*, *renal failure*, *VO*_2_*max < 10 mL/Kg*/*min*, *ppoFEV*_1_ *or ppoDL*CO < *40%*, *systemic disease*, *or other risk factors*). *In these patients*, *multidisciplinary assessment is useful to consider different treatment options and select the best therapeutic approach*.

Level of evidence: Poor

Strength of recommendation: A

The preoperative evaluation and perioperative management of patients undergoing thoracic surgery requires a multidisciplinary approach to assess the relative risks and benefits of surgery, optimize perioperative conditions, and plan the treatment regimen. This approach is endorsed in multiple management guidelines (Brunelli et al. 2013; Brunelli et al. 2009; Della Rocca et al. 2016; Lim et al. 2010). The multidisciplinary team should include a thoracic surgeon specializing in lung cancer, a medical oncologist, a radiation oncologist, a pulmonologist, and an anesthesiologist (Brunelli et al. 2013). A multidisciplinary approach to management may be particularly useful in patients who are borderline candidates for surgery (Brunelli et al. 2013).

### Preparation

Recommendation 13: *We recommend preoperative exercise rehabilitation* (*prehabilitation*) *in candidates for curative surgical intervention for lung cancer as it may reduce postoperative pulmonary complications*. *Since prehabilitation may reduce length of stay and postoperative pulmonary complications*, *it may be useful in COPD patients with mild to severe airway obstruction*. *Multimodal prehabilitation* (*early functional respiratory evaluation*, *smoking cessation*, *respiratory rehabilitation*, *nutritional status*, *physical exercise*) *is more effective than unimodal prehabilitation*. *It is advisable to schedule a preoperative prehabilitation program of 3 weeks*.

Level of evidence: Poor

Strength of recommendation: A

The term prehabilitation refers to preoperative physical conditioning intended to enhance the patient’s capacity to withstand the stress of surgery and promote postoperative recovery (Batchelor et al. 2019). Studies in patients undergoing pulmonary resection for lung cancer have shown that prehabilitation regimens improve measures of lung function such as FEV_1_, forced vital capacity, and performance in the 6-min walk test (Cavalheri and Granger 2017; Morano et al. 2013; Sebio Garcia et al. 2016; Ni et al. 2017; Pouwels et al. 2015; Vagvolgyi et al. 2017), and hence prehabilitation is now recommended in guidelines for a variety of thoracic surgery procedures (Batchelor et al. 2019; Mahendran and Naidu 2018; Tew et al. 2018). There is also evidence that prehabilitation is associated with lower rates of postoperative pulmonary complications, and shorter hospital stays, in patients undergoing resection for lung cancer (Cavalheri and Granger 2017; Sebio Garcia et al. 2016; Ni et al. 2017; Pouwels et al. 2015; Benzo et al. 2011; Boujibar et al. 2018; Steffens et al. 2018). For example, in a 2017 Cochrane review, the risk of postoperative pulmonary complications was reduced by 67% (risk ratio [RR] 0.33, 95% CI 0.17–0.61), compared with non-exercise groups; however, caution is needed when interpreting such findings because of marked differences in study design, the risk of bias, and small sample sizes in many trials (Cavalheri and Granger 2017). There is some evidence that reductions in pulmonary complications following prehabilitation are confined to patients with poor preoperative lung function (Batchelor et al. 2019). However, the majority of trials included in systematic reviews and meta-analyses have included mainly patients with mild to moderate pulmonary impairment prior to surgery: only a few studies have included patients with more pronounced respiratory impairment. One recent systematic review has specifically examined the use of prehabilitation in frail surgical patients (not specifically thoracic surgery) (Milder et al. 2018). This review found no evidence of improved postoperative functional recovery in patients undergoing prehabilitation: although reductions in mortality and duration of hospitalization were reported in some studies, the quality of the available evidence was low.

The prehabilitation protocols used in different studies vary markedly, both in intensity and duration (Benzo et al. 2011; Pehlivan et al. 2011; Stefanelli et al. 2013). The exercise protocol should be clearly defined in terms of the number of exercise sessions per day, and the number of days on which sessions are held; importantly, a self-managed protocol cannot be considered effective unless the exercise is supervised.

In addition to exercise, prehabilitation regimens in thoracic surgery patients may also encompass other interventions, such as optimizing concomitant medical conditions and nutritional status and smoking cessation (Mahendran and Naidu 2018). One study in patients undergoing resection for lung cancer has investigated the efficacy of a prehabilitation program of exercise, smoking cessation, and optimization of medical therapy, but found no significant improvement in respiratory function from baseline levels (Bobbio et al. 2008). By contrast, a further study found a significant improvement in respiratory function following implementation of a prehabilitation program of exercise and smoking cessation in COPD patients undergoing elective surgery (Vagvolgyi et al. 2017). Notwithstanding these conflicting results, a multidisciplinary approach remains indispensable in both short-term and long-term rehabilitation, but at present there is insufficient evidence of effectiveness to support measures other than exercise for prehabilitation in thoracic surgery patients. This situation with the experience in other surgical specialties, in which resolution of anemia and malnutrition are important elements of prehabilitation programs (Tew et al. 2018).

Recommendation 14: *Patients*’ *engagement has proven benefits on both clinical outcomes and healthcare sustainability*. *We suggest a Patient Health Engagement* (*PHE*) *model to monitor patients*’ *engagement and psychological needs and expectations*.

Level of evidence: Fair

Strength of recommendation: B

There is evidence that involving the patient in their care by the provision of preoperative counseling may reduce fear and postoperative fatigue and pain, enhance recovery, and facilitate early discharge from hospital (Batchelor et al. 2019). For this reason, we suggest a course of care that includes the use of a Patient Health Engagement (PHE) model to measure the level of engagement; in addition, education about the procedure should be provided to family members, because there is evidence that engagement of both the patient and family members contributes to improved postoperative outcomes (Graffigna and Barello 2018).

A course of treatment is suggested that focuses on patient empowerment by establishing a pre- and postoperative rehabilitation program in order to improve the effectiveness of ERAS protocols, and by encouraging patients to be actively involved in this program (Schatz 2015; Taurchini et al. 2018). This is particularly important in older patients, who are less likely to engage with ERAS programs than younger patients (Schatz 2015): since ERAS protocols have been shown to be effective in reducing complications in thoracic surgery patients (Graffigna and Barello 2018; Schatz 2015; Taurchini et al. 2018; Dumans-Nizard et al. 2016), lower rates of engagement may place older patients at higher risk of postoperative complications.

It should be noted that, although patient engagement and provision of information to patients’ families improves postoperative outcomes and allows quicker discharge from hospital and return to work (Batchelor et al. 2019; Graffigna and Barello 2018; Schatz 2015), there is currently no consensus about the optimum modality, intensity, and timing of preoperative rehabilitation.

## Preoperative care

Recommendation 15: *We recommend the video*-*assisted thoracoscopic approach for lung surgery whenever possible*, *because of the lower incidence of postoperative complications*, *shorter length of hospital stay*, *and lower levels of postoperative pain associated with this technique*.

Level of evidence: Good

Strength of recommendation: A

There is consistent evidence that the use of VATS decreases postoperative complications and shortens the duration of hospitalization, compared with open pneumonectomy or lobectomy (Cao et al. 2013; Greenwood and West 2013; Linden et al. 2014; Mohiuddin and Swanson 2013; Paul et al. 2010; Santambrogio et al. 1995; Whitson et al. 2008). For example, in a systematic review of 39 studies, VATS lobectomy was associated with a shorter duration of chest tube drainage, compared with open thoracotomy (4.2 vs. 5.7 days, respectively, *P* = 0.025), a shorter LOS (mean 8.3 vs. 13.3 days, *P* = 0.016), and a higher 4-year survival rate (88.4% vs. 71.4%, *P* = 0.003) (Whitson et al. 2008). In a study in which propensity score matching was used to adjust for differences in baseline characteristics, VATS lobectomy was associated with significant reductions, compared with open lobectomy, in postoperative arrhythmias (7.3% vs. 11.5%, respectively, *P* = 0.0004), reintubation (1.4% vs. 3.1%, *P* = 0.0046), blood transfusion (2.4% vs. 4.7%, *P* = 0.0028), LOS (median 4.0 vs. 6.0 days, *P* < 0.0001), and chest tube duration (median 3.0 vs. 4.0 days, *P* < 0.0001) (Paul et al. 2010). A subsequent meta-analysis of propensity score-matched patients enrolled in four studies found that, compared with open thoracotomy, VATS was associated with significantly fewer overall complications, significantly lower rates of prolonged air leak, pneumonia, atrial arrhythmias and renal failure, and a shorter LOS (Cao et al. 2013).

In addition to reducing postoperative complications, VATS has also been shown to reduce postoperative pain and related morbidity (Trinh et al. 2016; Mohiuddin and Swanson 2013; Bendixen et al. 2016; Giudicelli et al. 1994; Hazelrigg et al. 2002; Landreneau et al. 1993; Mahtabifard et al. 2007; Sedrakyan et al. 2004). In a systematic review of 12 randomized trials in patients undergoing lung resection or treatment of pneumothorax, VATS procedures were associated with lower pain scores, and less use of analgesic medication, compared with open thoracotomy (Sedrakyan et al. 2004). More recently, a randomized controlled trial involving 206 patients undergoing lobectomy for early stage lung cancer found that, compared with open thoracotomy, VATS was associated with significant reductions in the proportion of patients with moderate-to-severe pain during 52 weeks of follow-up, and the proportion of patients with clinically relevant pain during the 24 h after surgery (Bendixen et al. 2016).

The choice of surgical strategy (VATS or open thoracotomy) should be established during the preoperative evaluation, and shared with all members of the care team ahead of surgery.

Recommendation 16: *We do not recommend preoperative mechanical bowel preparation in patients undergoing lung surgery.*

Level of evidence: Poor

Strength of recommendation: D

A retrospective study of 560 thoracic surgery patients showed that preoperative mechanical bowel preparation did not reduce postoperative complications or length of stay (Yamazaki et al. 2004). This procedure is not recommended by the ERAS guidelines for colon surgery (Gustafsson et al. 2013).

Recommendation 17: *We recommend limiting clear fluid and solid fasting up to 2 and 6 h*, *respectively, in patients undergoing lung surgery who are not at risk of delayed gastric emptying*.

Level of evidence: Good

Strength of recommendation: A

In a randomized controlled trial, patients undergoing thoracic surgery according to a fast track pathway in which clear fluids were permitted until 2 h before induction of anesthesia had fewer postoperative complications than patients who were required to fast for 6 h prior to surgery (6.6% vs. 35%, respectively, *P* = 0.009) (Muehling et al. 2008). Guidelines in a number of surgical specialties endorse preoperative fasting intervals of 2 h for clear fluids and 6 h for solid foods (Batchelor et al. 2019; Gustafsson et al. 2013; Smith et al. 2011).

Recommendation 18: *We recommend preoperative carbohydrate loading with clear fluids up to 2 h prior to surgery for patients undergoing lung surgery*, *especially malnourished patients*, *in order to reduce perioperative discomfort and insulin resistance*.

Level of evidence: Poor

Strength of recommendation: A

Preoperative carbohydrate loading reduces perioperative discomfort and insulin resistance, and is feasible and well tolerated in patients undergoing thoracic surgery (Kerr et al. 2017; Zakeri et al. 2015). However, the impact of carbohydrate loading on postoperative outcomes in patients undergoing thoracic surgery remains to be evaluated in randomized controlled trials (Kerr et al. 2017). Preoperative carbohydrate loading before major surgery is recommended in guidelines published by the ERAS Society (Ljungqvist et al. 2017) and the European Society for Clinical Nutrition and Metabolism (ESPEN) (Weimann et al. 2017).

The 2019 ERAS/ESTS lung surgery guidelines note that there is insufficient evidence to recommend the preoperative use of immune-enhancing nutrition, but this may have a role in the postoperative management of malnourished patients (Batchelor et al. 2019).

Recommendation 19: *We suggest avoiding the routine use of benzodiazepines before thoracic surgery*, *especially in elderly people*. *When used in selected cases*, *short*-*acting benzodiazepines should be preferred over long*-*acting agents*.

Level of evidence: Fair

Strength of recommendation: B

Anxiolytic agents such as benzodiazepines are widely used prior to induction of anesthesia. In general, short-acting agents such as midazolam are preferred to long-acting agents because the latter are associated with delayed postoperative recovery (Umari et al. 2018). However, midazolam has itself been shown to be associated with late discharge from the post-anesthesia care unit (PACU), and lower scores on psychomotor performance tests (Umari et al. 2018). In a retrospective review of approximately 6000 patients undergoing general endotracheal anesthesia, implementation of an anesthetic protocol with reduced midazolam usage, designed to reduce the duration of postoperative hospitalization by decreasing residual sedation and postoperative nausea and vomiting, was associated with a significant (*P* < 0.001) reduction in median recovery time, from 72 min (interquartile range [IQR] 50–102) to 62 min (IQR 44–90) (Weingarten et al. 2015).

The European Society of Anaesthesiologists (ESA) guideline on postoperative delirium discourages the use of benzodiazepine premedication, except for patients with severe anxiety (Aldecoa et al. 2017). One randomized trial has found that lorazepam administration prolongs extubation time and post-anesthesia recovery, compared with no premedication or placebo, without any beneficial effect on perioperative discomfort (Maurice-Szamburski et al. 2015). However, specific data on the preoperative use of benzodiazepines are lacking in thoracic surgery.

## Conclusions

The PACTS group has sought to identify critical issues in the preoperative, care of patients undergoing lung resection, and to provide appropriate guidance. Wherever possible, our recommendations are based on good-quality supporting evidence: where such evidence is limited, the recommendations are framed as suggestions or possibilities for consideration.

Many of the recommendations apply equally to VATS and open surgery. While we recognize that the use of VATS is increasing (indeed, we recommend this approach wherever possible), it is important to recognize that open thoracotomy is by no means an obsolete procedure, and may be more appropriate for certain patients than VATS. Our literature reviews and discussions highlighted that a comprehensive risk assessment prior to surgery is essential in candidates for lung resection, as this will help to determine the optimal surgical approach (i.e., open thoracotomy or VATS) in the individual patient. It is hoped that this approach will contribute to achieving optimal postoperative outcomes in the greatest number of thoracic surgery patients.

The ERAS lung surgery guidelines (Batchelor et al. 2019) were published while our recommendations were in development. We believe that these recommendations extend and complement those of the ERAS guidelines for a number of reasons. First, they cover a number of aspects of the preoperative evaluation that are not addressed in the ERAS guidelines, notably the cardiovascular assessment and the use of spirometry and CPET. Second, as described in an accompanying paper, aspects of anesthesiologic care such as depth of anesthesia monitoring, neuromuscular blockade, and hemodynamic monitoring are covered in greater detail than in the ERAS guidelines. Finally, our recommendations focus specifically on elective surgery for lung cancer.

The management of thoracic surgery patients is a rapidly changing field, in which strongly evidence-based recommendations have hitherto been lacking. Further refinement of our recommendations can be anticipated as the literature continues to evolve.

## Supplementary Information

**Additional file 1.** Search strategy and keywords.

## Data Availability

Data sharing is not applicable to this article as no datasets were generated or analyzed during the current study.

## Abbreviations

AIPO: Italian Association of Hospital Pneumologists (Associazione Italiana Pneuomologi Ospedalieri)
AKI: Acute kidney injury
ASA: American Society of Anesthesiologists
CI: Confidence interval
COPD: Chronic obstructive pulmonary disease
CPET: Cardiopulmonary exercise testing
DLCO: Carbon monoxide diffusing capacity
eGFR: Estimated glomerular filtration rate
ERAS: Enhanced recovery after surgery
ESA: European Society of Anaesthesiologists
ESPEN: European Society for Clinical Nutrition and Metabolism
ESTS: European Society of Thoracic Surgeons
FEV_1_: Forced expiratory volume in 1 second
HR: Hazard ratio
ICU: Intensive care unit
IQR: Interquartile range
KDIGO: Kidney Disease Improving Global Outcomes
LOS: Length of stay
NRI: Nutritional Risk Index
NSQIP: American College of Surgeons National Surgical Quality Improvement Program
OR: Odds ratio
OSA: Obstructive sleep apnea
PaCO_2_: Partial pressure of carbon dioxide
PACTS: Perioperative Anesthesia Care in Thoracic Surgery
PACU: Post-anesthesia care unit
PaO_2_: Partial pressure of arterial oxygen
PHE: Patient Health Engagement
PICO: Patients, intervention, comparison, outcome
ppoDLCO: Predicted postoperative DLCO
ppoFEV_1_: Predicted postoperative FEV_1_
RCRI: Revised Cardiac Risk Index
RR: Risk ratio
SEER: Surveillance, Epidemiology, and End Results
SIAARTI: Italian Society of Anesthesia, Analgesia, Resuscitation, and Intensive Care (Società Italiana di Anestesia Analgesia Rianimazione e Terapia Intensiva)
SIC: Italian Society of Surgery (Società Italiana di Chirurgia)
SICT: Italian Society of Thoracic Surgery (Società Italiana di Chirurgia Toracica)
SIET: Italian Society of Thoracic Endoscopy (Società Italiana di Endoscopia Toracica)
SIP/IRS: Italian Society of Pneumology (Società Italiana di Pneumologia)
USPFTF: United States Preventative Services Task Force
VATS: Video-assisted thoracic surgery
VO_2_max: Maximum oxygen consumption
