# Supplementary material for: Recommendations from the Italian intersociety consensus on Perioperative Anesthesia Care in Thoracic surgery (PACTS) part 1: preadmission and preoperative care
Source: Perioper Med (Lond). 2020 Dec 1;9:37. doi: 10.1186/s13741-020-00168-y (PMC7704118; doi:10.1186/s13741-020-00168-y)
Supplement: Supplementary file 1 — Additional file 1. Search strategy and keywords. [file 13741_2020_168_MOESM1_ESM.docx]

Supplementary material

**Search strategy and keywords**

**Thoracic surgery literature strategies**

All searches were confined to English and Italian language papers. No restrictions on date were applied.

**Preoperative phase**

***Risk evaluation (PONV, TVP, FA, respiratory risk)***

*Pubmed:*

- (lung resection) AND ((Aged[MeSH Terms]) OR (Aged, 80 and over[MeSH Terms])) AND (death OR (fatal outcome) OR pneumonia OR (intensive care units) OR (oxygen inhalation therapy)) AND adults
- (lung resection) AND (smoking OR (smoking, tobacco) OR (smoking cessation) OR (cigarette smoking)) AND (death OR (fatal outcome) OR pneumonia OR (intensive care units) OR (oxygen inhalation therapy)) AND adults
- (lung resection) AND (ASA class) AND (death OR (fatal outcome) OR pneumonia OR (intensive care units) OR (oxygen inhalation therapy)) AND Adults^[[1]](#footnote-1)^
- (lung resection) AND (obesity OR (body weight) OR (Pulmonary disease, chronic obstructive) OR (lung diseases, obstructive) OR (respiratory insufficiency) OR (Forced expiratory volume) OR (lung volume measurements)) AND (death OR (fatal outcome) OR pneumonia OR (intensive care units) OR (oxygen inhalation therapy)) AND adults
- Pneumonectomy AND ((Forced expiratory volume) OR (lung volume measurements) OR (respiratory function tests)) AND (data accuracy) AND (Scintigraphy OR (radionuclide imaging/methods)) AND adults^*^
- (((((((((((Lung[MeSH Major Topic]) AND resection) OR Pulmonary surgical procedures[MeSH Major Topic]) AND stair climbing[MeSH Major Topic]) AND data accuracy[MeSH Terms]) OR dimensional measurement accuracy[MeSH Terms]) AND cardiopulmonary exercise test[MeSH Major Topic]) AND Humans[Mesh])) OR endurance shuttle walk test[MeSH Major Topic]) OR incremental shuttle walk test[MeSH Major Topic]) OR 6-minute walk test[MeSH Major Topic] AND adults
- (((Pneumonectomy[MeSH Terms]) OR (Pulmonary surgical procedures[MeSH Terms])) AND ((aged[MeSH Terms]) OR (aged, 80 and over[MeSH Terms])) AND (death[MeSH Terms] OR (fatal outcome[MeSH Terms]) OR pneumonia[MeSH Terms] OR (intensive care units[MeSH Terms]) OR (oxygen inhalation therapy[MeSH Terms])) AND adults
- (((Pneumonectomy[MeSH Terms]) OR (Pulmonary surgical procedures[MeSH Terms])) AND (smoking OR (smoking, tobacco) OR (smoking cessation) OR (cigarette smoking)) AND (death[MeSH Terms] OR (fatal outcome[MeSH Terms]) OR pneumonia[MeSH Terms] OR (intensive care units[MeSH Terms]) OR (oxygen inhalation therapy[MeSH Terms])) AND adults
- (((Pneumonectomy[MeSH Terms]) OR (Pulmonary surgical procedures[MeSH Terms])) AND (obesity OR (body weight)) AND (death[MeSH Terms] OR (fatal outcome[MeSH Terms]) OR pneumonia[MeSH Terms] OR (intensive care units[MeSH Terms]) OR (oxygen inhalation therapy[MeSH Terms])) AND adults
- (((Pneumonectomy[MeSH Terms]) OR (Pulmonary surgical procedures[MeSH Terms]) OR (lung volume reduction[MESH Terms)) AND ((cardiac evaluation) OR (electrocardiography[MESH terms])) AND (death[MeSH Terms] OR (fatal outcome[MeSH Terms]) OR pneumonia[MeSH Terms] OR (intensive care units[MeSH Terms]) OR (oxygen inhalation therapy[MeSH Terms])) AND adults

*Embase^[[2]](#footnote-2)^:*

- 'adults' AND ('lung resection'/mj OR 'lung resection' OR 'lung volume reduction surgery' OR 'lung, resected' OR 'pneumectomy' OR 'pneumonectomy' OR 'pneumonic resection' OR 'pneumoresection' OR 'pulmonary resection' OR 'pulmonectomy' OR 'resected lung' OR 'resection, lung' OR 'surgery, lung volume reduction') AND ('aged'/mj OR 'aged' OR 'elderly') AND 'younger patients' AND (death OR pneumonia OR 'intensive care unit' OR 'oxygen therapy')
- 'adults' AND ('lung resection'/mj OR 'lung resection' OR 'lung volume reduction surgery' OR 'lung, resected' OR 'pneumectomy' OR 'pneumonectomy' OR 'pneumonic resection' OR 'pneumoresection' OR 'pulmonary resection' OR 'pulmonectomy' OR 'resected lung' OR 'resection, lung' OR 'surgery, lung volume reduction') AND ('smoking'/mj OR 'smoker' OR 'smokers' OR 'smoking' OR 'tobacco smoking') AND ('death'/mj OR 'death' OR 'mors' OR 'pneumonia' OR 'intensive care unit' OR 'oxygen therapy')
- 'adult' AND ('lung resection'/mj OR 'lung resection' OR 'lung volume reduction surgery' OR 'lung, resected' OR 'pneumectomy' OR 'pneumonectomy' OR 'pneumonic resection' OR 'pneumoresection' OR 'pulmonary resection' OR 'pulmonectomy' OR 'resected lung' OR 'resection, lung' OR 'surgery, lung volume reduction') AND 'asa class'
- 'adults' AND ('lung resection'/mj OR 'lung resection' OR 'lung volume reduction surgery' OR 'lung, resected' OR 'pneumectomy' OR 'pneumonectomy' OR 'pneumonic resection' OR 'pneumoresection' OR 'pulmonary resection' OR 'pulmonectomy' OR 'resected lung' OR 'resection, lung' OR 'surgery, lung volume reduction') AND 'obesity'/mj
- ('lung resection'/mj OR 'lung resection' OR 'lung volume reduction surgery' OR 'lung, resected' OR 'pneumectomy' OR 'pneumonectomy' OR 'pneumonic resection' OR 'pneumoresection' OR 'pulmonary resection' OR 'pulmonectomy' OR 'resected lung' OR 'resection, lung' OR 'surgery, lung volume reduction') AND ('chronic obstructive lung disease'/mj OR 'chronic airflow obstruction' OR 'chronic airway obstruction' OR 'chronic obstructive bronchitis' OR 'chronic obstructive bronchopulmonary disease' OR 'chronic obstructive lung disease' OR 'chronic obstructive lung disorder' OR 'chronic obstructive pulmonary disease' OR 'chronic obstructive pulmonary disorder' OR 'chronic obstructive respiratory disease' OR 'copd' OR 'lung chronic obstructive disease' OR 'lung disease, chronic obstructive' OR 'lung diseases, obstructive' OR 'obstructive lung disease' OR 'obstructive lung disease, chronic' OR 'obstructive pulmonary disease' OR 'obstructive respiratory disease' OR 'obstructive respiratory tract disease' OR 'pulmonary disease, chronic obstructive' OR 'pulmonary disorder, chronic obstructive') AND normal AND ('death' OR 'pneumonia'/mj OR 'intensive care unit' OR 'oxygen therapy')
- ('lung resection'/mj OR 'lung resection' OR 'lung volume reduction surgery' OR 'lung, resected' OR 'pneumectomy' OR 'pneumonectomy' OR 'pneumonic resection' OR 'pneumoresection' OR 'pulmonary resection' OR 'pulmonectomy' OR 'resected lung' OR 'resection, lung' OR 'surgery, lung volume reduction') AND ('lung function'/mj OR 'function, lung' OR 'lung function' OR 'pulmonary function' OR 'regional lung function') AND normal AND ('death'/mj OR 'pneumonia' OR 'intensive care unit' OR 'oxygen therapy')
- ('lung resection'/mj OR 'lung resection' OR 'lung volume reduction surgery' OR 'lung, resected' OR 'pneumectomy' OR 'pneumonectomy' OR 'pneumonic resection' OR 'pneumoresection' OR 'pulmonary resection' OR 'pulmonectomy' OR 'resected lung' OR 'resection, lung' OR 'surgery, lung volume reduction') AND ('lung function'/mj OR 'function, lung' OR 'lung function' OR 'pulmonary function' OR 'regional lung function') AND normal AND ('death'/mj OR 'pneumonia' OR 'intensive care unit' OR 'oxygen therapy')
- ('lung resection'/mj OR 'lung resection' OR 'lung volume reduction surgery' OR 'lung, resected' OR 'pneumectomy' OR 'pneumonectomy' OR 'pneumonic resection' OR 'pneumoresection' OR 'pulmonary resection' OR 'pulmonectomy' OR 'resected lung' OR 'resection, lung' OR 'surgery, lung volume reduction') AND ('lung volume'/mj OR 'lung volume' OR 'lung volume measurements' OR 'pulmonary volume' OR 'respiratory volume' OR 'volume, lung') AND normal AND ('death' OR 'pneumonia'/mj OR 'intensive care unit' OR 'oxygen therapy')
- ('lung resection'/mj OR 'lung resection' OR 'lung volume reduction surgery' OR 'lung, resected' OR 'pneumectomy' OR 'pneumonectomy' OR 'pneumonic resection' OR 'pneumoresection' OR 'pulmonary resection' OR 'pulmonectomy' OR 'resected lung' OR 'resection, lung' OR 'surgery, lung volume reduction') AND 'cardiac evaluation'
- ('lung resection'/mj OR 'lung resection' OR 'lung volume reduction surgery' OR 'lung, resected' OR 'pneumectomy' OR 'pneumonectomy' OR 'pneumonic resection' OR 'pneumoresection' OR 'pulmonary resection' OR 'pulmonectomy' OR 'resected lung' OR 'resection, lung' OR 'surgery, lung volume reduction' OR 'emphysema'/mj) AND 'cancer surgery'/mj AND ('death'/mj OR 'pneumonia'/mj OR 'intensive care unit'/mj OR 'oxygen therapy'/mj OR 'o2 administration' OR 'o2 therapy' OR 'oxygen administration' OR 'oxygen inhalation therapy' OR 'oxygen insufflation' OR 'oxygen therapy' OR 'oxygen treatment')

***Postoperative assistance***

*Pubmed:*

- ((Pneumonectomy OR lobectomy) AND (lung resection) AND ((Nursing, Post-Anesthesia) OR (Nursing, Post-Surgical) OR (Post-Anesthesia Nursing) OR (Post-Surgical Nursing)) AND adults
- ((Pneumonectomy OR lobectomy) AND (lung resection) AND (intensive care units)) AND adults
- ((lung resection) AND ((lung volume measurements) OR (forced expiratory flow rates) OR (respiratory function tests)) AND ((intensive care units) OR (Nursing, Post-Anesthesia) OR (Nursing, Post-Surgical) OR (Post-Anesthesia Nursing) OR (Post-Surgical Nursing)) AND adults
- ((lung resection) AND (diabetes OR creatinine) AND ((intensive care units) OR (Nursing, Post-Anesthesia) OR (Nursing, Post-Surgical) OR (Post-Anesthesia Nursing) OR (Post-Surgical Nursing))) AND adults
- ((lung resection) AND ((heart diseases) OR (cardiac diseases) OR (arrhythmias, cardiac) OR (atrial fibrillation)) AND ((intensive care units) OR (Nursing, Post-Anesthesia) OR (Nursing, Post-Surgical) OR (Post-Anesthesia Nursing) OR (Post-Surgical Nursing))) AND adults
- ((lung resection) AND ((lung volume measurements) OR (forced expiratory flow rates) OR (respiratory function tests)) AND ((exercise test) OR (Bicycle Ergometry Test) OR (Cardiopulmonary Exercise Test) OR (Fitness Testing) OR (Physical Fitness Testing) OR (Step Test) OR (Stress Test) OR (Treadmill Test)) AND ((intensive care units) OR (Nursing, Post-Anesthesia) OR (Nursing, Post-Surgical) OR (Post-Anesthesia Nursing) OR (Post-Surgical Nursing)) AND adults

*Embase:*

- 'pneumonectomy' AND 'lung resection' AND ('recovery room'/exp OR 'intensive care unit'/exp OR 'postoperative care'/exp)
- pneumonectomy AND ('lung function test'/exp OR 'lung volume'/exp) AND 'lung resection'/exp AND ('postoperative care'/exp OR 'care, postoperative' OR 'postoperative care' OR 'postoperative therapy' OR 'postoperative treatment')
- 'lung resection'/exp AND ('diabetes mellitus'/exp OR 'creatinine blood level'/exp OR 'blood creatinine' OR 'creatinine blood level' OR 'creatinine, serum' OR 'plasma creatinine' OR 'serum creatinine') AND ('postoperative care'/exp OR 'care, postoperative' OR 'postoperative care' OR 'postoperative therapy' OR 'postoperative treatment')
- 'lung resection'/exp AND (('heart disease'/exp OR 'cardiac anomaly' OR 'cardiac disease' OR 'cardiac disturbance' OR 'cardiopathy' OR 'heart deficiency' OR 'heart deformity' OR 'heart disease' OR 'heart diseases' OR 'heart disorder' OR 'heart dysfunction') AND 'cardiac arrhythmias' OR 'atrial fibrillation'/exp) AND ('postoperative care'/exp OR 'care, postoperative' OR 'postoperative care' OR 'postoperative therapy' OR 'postoperative treatment')
- 'lung resection'/exp AND ('cardiopulmonary exercise test'/exp OR 'cardiopulmonary exercise test' OR 'walk test'/exp OR 'stair climbing test'/exp OR 'exercise test'/exp OR 'shuttle walk test'/exp) AND ('postoperative care'/exp OR 'care, postoperative' OR 'postoperative care' OR 'postoperative therapy' OR 'postoperative treatment')
- 'lung resection'/exp AND ('aged'/exp OR 'very elderly'/exp) AND 'younger patients' AND ('postoperative care'/exp OR 'care, postoperative' OR 'postoperative care' OR 'postoperative therapy' OR 'postoperative treatment')

***Prehabilitation***

*Pubmed:*

- (((lung resection) OR (pneumonectomy)) AND (rehabilitation) AND ((postoperative complications) OR (complication, postoperative))
- ((lung resection) OR (pneumonectomy)) AND (rehabilitation) AND ((cardiopulmonary exercise test) OR (respiratory failure))
- ((lung resection) OR (pneumonectomy)) AND ((pulmonary disease, chronic obstructive) OR (lung diseases, obstructive)) AND (rehabilitation)
- ((lung resection) OR (pneumonectomy)) AND ((lung function test) OR (pulmonary function test)) AND (rehabilitation))
- ((lung resection) OR (pneumonectomy)) AND (home care) AND (rehabilitation))

*Embase:*

- 'thoracic surgery' AND 'rehabilitation'/mj AND ('postoperative complication'/exp OR 'complication, postoperative' OR 'complication, surgical' OR 'post-operative complication' OR 'post-operative complications' OR 'postoperative complication' OR 'postoperative complications' OR 'postsurgical complication' OR 'surgical complication')
- 'thoracic surgery' AND 'rehabilitation'/mj AND ('respiratory failure'/exp OR 'deficiency, respiration' OR 'respiration deficiency' OR 'respiration disturbance' OR 'respiration failure' OR 'respiration insufficiency' OR 'respiratory deficiency' OR 'respiratory disturbance' OR 'respiratory dysfunction' OR 'respiratory failure' OR 'respiratory insufficiency' OR 'respiratory tract insufficiency' OR 'cardiopulmonary exercise test'/exp OR 'cardiopulmonary exercise test')^[[3]](#footnote-3)^
- 'thoracic surgery' AND ('chronic obstructive lung disease'/exp OR 'chronic obstructive pulmonary disease' OR 'copd' OR 'lung disease, chronic obstructive' OR 'obstructive lung disease, chronic' OR 'pulmonary disease, chronic obstructive') AND 'rehabilitation'/exp
- 'thoracic surgery' AND 'rehabilitation'/exp AND ('lung function test'/exp OR 'function test, lung' OR 'function test, pulmonary' OR 'lung function test' OR 'pulmonary function test' OR 'respiratory function test' OR 'respiratory function tests' OR 'respiratory test' OR 'ventilation test')
- 'thoracic surgery' AND 'rehabilitation'/exp AND ('home care'/exp OR 'domestic health care' OR 'domiciliary care' OR 'home care' OR 'home care agencies' OR 'home care program' OR 'home care programme' OR 'home care service' OR 'home care services' OR 'home care services, hospital-based' OR 'home health care' OR 'home health nursing' OR 'home help' OR 'home nursing' OR 'home service' OR 'home treatment' OR 'homecare' OR 'homemaker services')

***Personalized plan for anaesthesiology***

*Pubmed:*

- (((lung resection) OR (pneumonectomy)) AND (smoking cessation) AND ((postoperative complications) OR (complication, postoperative)))
- (((lung resection) OR (pneumonectomy)) AND (benzodiazepine) AND ((postoperative complications) OR (complication, postoperative)))
- ((thoracic surgery) AND (benzodiazepine) AND ((postoperative complications) OR (complication, postoperative)))
- (((lung resection) OR (pneumonectomy)) AND (gabapentin) AND ((postoperative pain) OR (analgesic use) OR (postoperative complications) OR (complication, postoperative)))
- (((Surgery, Thoracic, Video-Assisted) OR (Video-Assisted Thoracic Surgery) OR (Video-Assisted Thoracoscopic Surgery)) AND ((Thoracotomy) AND ((postoperative complications) OR (complication, postoperative) OR ((postoperative pain) OR (analgesic use)))
- ((((thoracic surgery) AND ((bronchoscopy) OR (Bronchoscopic Surgery) OR (Bronchoscopic Surgical Procedures) OR (Surgery, Bronchoscopic) OR (Surgical Procedures, Bronchoscopic))) AND (tube positioning)))
- (((lung resection) OR (pneumonectomy)) AND ((Analgesia, Patient-Controlled) OR (analgesia, epidural) OR (intravenous anesthetics) OR (morphines)))

*Embase:*

- 'thoracic surgery' AND ('smoking cessation'/exp OR 'gabapentin'/exp OR benzodiazepines OR 'video-assisted thorascopic surgery') AND ('postoperative complications' OR 'recovery time'/exp)
- 'thoracic surgery' AND ('bronchoscopy'/exp OR 'fibreoptic bronchoscopy') AND 'tube positioning'
- 'thoracic surgery' AND ('patient controlled analgesia'/exp OR 'analgesia, patient controlled' OR 'analgesia, patient-controlled' OR 'patient controlled anaesthesia' OR 'patient controlled analgesia' OR 'patient controlled anesthesia' OR 'self anaesthesia' OR 'self anesthesia' OR 'epidural analgesia'/exp OR 'analgesia, epidural' OR 'epidural analgesia' OR 'extradural analgesia' OR 'peridural analgesia' OR 'intravenous analgesia' OR 'inhalation analgesia' OR 'morphine'/exp) AND 'postoperative complications'

***Fasting and nutrition***

*Pubmed:*

- (((thoracic surgery) OR (lung resection) OR (pneumonectomy)) AND ((malnutrition) OR (protein-calorie malnutrition) OR (malnutrition universal screening tool) OR (hypoalbuminemia)) AND ((postoperative complications) OR (complication, postoperative)))^[[4]](#footnote-4)^
- (((thoracic surgery) OR (lung resection) OR (pneumonectomy)) AND (bowel preparation) AND ((postoperative complications) OR (complication, postoperative)))
- (((thoracic surgery) OR (lung resection) OR (pneumonectomy)) AND (fasting) AND ((postoperative complications) OR (complication, postoperative)))
- (((thoracic surgery) OR (lung resection) OR (pneumonectomy)) AND ((dietary supplements) OR (carbohydrate loading)) AND ((postoperative complications) OR (complication, postoperative) OR (hospitalization)))

*Embase:*

- ('thoracic surgery' OR 'lung resection'/exp OR pneumonectomy) AND ('malnutrition'/exp OR 'deficient nutrition' OR 'malnourishment' OR 'malnutrition' OR 'severe acute malnutrition' OR 'underfeeding' OR 'undernourishment' OR 'undernutrition' OR 'must score' OR 'hypoalbuminemia'/exp) AND ('postoperative complications' OR 'duration of hospitalization')
- ('thoracic surgery' OR 'lung resection'/exp OR pneumonectomy) AND ('intestine preparation'/exp OR 'bowel preparation' OR 'intestine preparation') AND ('postoperative complications' OR 'duration of hospitalization')^[[5]](#footnote-5)^
- ('thoracic surgery' OR 'lung resection'/exp OR pneumonectomy) AND fasting AND ('postoperative complications' OR 'duration of hospitalization')
- 'thoracic surgery' OR 'lung resection'/exp OR pneumonectomy) AND ('carbohydrate loading diet'/exp OR 'carbohydrate loading' OR 'carbohydrate loading diet' OR 'diet, carbohydrate loading' OR 'immune-enhancing nutritional supplements') AND ('postoperative complications' OR 'duration of hospitalization')

***Patient engagement***

*Pubmed:*

- (((thoracic surgery) OR (lung resection) OR (pneumonectomy)) AND ((preoperative) AND (psychological) AND ((counseling) OR (support))) AND ((postoperative complications) OR (complication, postoperative) OR (hospitalization)))
- (((thoracic surgery) AND ((aged) OR (aged, 80 and over)) AND (fast track protocol) AND ((morbidity) OR (mortality)))
- ((thoracic surgery) AND ((Patient Activation) OR (Patient Empowerment) OR (Patient Engagement) OR (Patient Involvement Patient Participation Rates)))

*Embase:*

- ('thoracic surgery' OR 'lung resection'/exp OR pneumonectomy) AND ('preoperative counselling' OR 'psychological preparation' OR 'psychological support'/exp) AND ('postoperative complications' OR 'duration of hospitalization')
- ('thoracic surgery' OR 'lung resection'/exp OR pneumonectomy) AND ('disease awareness' OR 'patient knowledge'/exp OR 'patient awareness' OR 'patient eduction') AND ('postoperative complication'/exp OR 'complication, postoperative' OR 'complication, surgical' OR 'post-operative complication' OR 'post-operative complications' OR 'postoperative complication' OR 'postoperative complications' OR 'postsurgical complication' OR 'surgical complication' OR 'duration of hospitalization')^[[6]](#footnote-6)^
- (('thoracic surgery' OR 'lung resection'/exp OR pneumonectomy) AND ('aged'/exp OR 'aged' OR 'aged patient' OR 'aged people' OR 'aged person' OR 'aged subject' OR 'elderly' OR 'elderly patient' OR 'elderly people' OR 'elderly person' OR 'elderly subject' OR 'senior citizen' OR 'senium') OR 'very elderly'/exp) AND 'fast track protocol' AND ('morbidity'/exp OR 'mortality'/exp OR 'excess mortality' OR 'mortality' OR 'mortality model')
- 'thoracic surgery' AND ('patient participation'/exp OR 'patient involvement' OR 'patient participation' OR 'patient participation rate')

**Intraoperative phase**

***Airways management***

*Pubmed:*

- (((lung resection) OR (pneumonectomy)) AND ((Bronchoscopy) OR (Bronchoscopic Surgery) OR (Bronchoscopic Surgical Procedures) OR (Surgery, Bronchoscopic) OR (Surgical Procedures, Bronchoscopic)) AND ((intubation) OR (intubation, endotracheal)))
- ((lung resection) AND ((intubation) OR (intubation, endotracheal))
- ((non-pulmonary thoracic surgery) OR (lung resection)) AND (one-lung ventilation))
- ((thoracic surgery) OR (lung resection)) AND (intubation, endotracheal) AND (placement))

*Embase:*

- 'lung resection'/exp AND ('bronchoscopy'/exp OR 'bronchial endoscopy' OR 'bronchoscopy' OR 'laryngotracheobronchoscopy' OR 'tracheobronchoscopy' OR 'fibreoptic bronchoscopy') AND ('auscultation'/exp OR 'auscultation')
- 'lung resection'/exp AND ('endotracheal intubation'/exp OR 'endotracheal intubation' OR 'intratracheal intubation' OR 'intubation, endotracheal' OR 'intubation, intratracheal' OR 'intubation, orotracheal' OR 'intubation, trachea' OR 'orotracheal intubation' OR 'trachea intubation' OR 'tracheal intubation' OR 'endobronchial intubation'/exp)
- ('lung resection'/exp OR 'non-pulmonary thoracic surgery') AND 'one lung ventilation'/exp
- ('lung resection'/exp OR 'non-pulmonary thoracic surgery') AND ('endotracheal intubation'/exp OR 'endobronchial intubation'/exp) AND placement

***Selection of drugs for anaesthesia***

*Pubmed:*

- ((thoracic surgery) AND ((benzodiazepines) OR (preanesthetic medication)) AND ((Endotracheal Extubation) OR (Extubation, Intratracheal) OR (Tracheal Extubation)))
- ((thoracic surgery) AND ((analgesics) AND (preanesthetic medication)) AND ((postoperative pain) OR (pain, postoperative)))
- (((thoracic surgery) AND ((Anesthetic Agents) OR (Anesthetic Drugs) OR (Anesthetic Effect) OR (Anesthetic Effects)) AND ((inflammation) OR (systemic inflammation) OR (alveolar inflammation)))
- (((thoracic surgery) AND (propofol) AND ((Anesthetic Agents) OR (Anesthetic Drugs)) AND ((respiratory complications) OR (postoperative complications) OR (complication, postoperative) OR (intraoperative complications)))
- (((thoracic surgery) AND (short-acting opioids) AND ((pulmonary vasoconstriction) OR (Endotracheal Extubation) OR (Extubation, Intratracheal) OR (Tracheal Extubation)))
- (((thoracic surgery) AND ((neuromuscular block) AND (corticosteroids) OR (sugammaedx)) AND ((pulmonary complications) OR (Postoperative complications) OR (complication, postoperative) OR (Endotracheal Extubation) OR (Extubation, Intratracheal) OR (Tracheal Extubation)))
- (((thoracic surgery) AND ((neuromuscular block) AND (corticosteroids) OR (sugammaedx)))
- ((thoracic surgery) AND (ketorolac))

*Embase:*

- 'thoracic surgery' AND ('premedication'/exp OR 'anaesthesia premedication' OR 'anaesthesia pretreatment' OR 'anesthesia premedication' OR 'anesthesia pretreatment' OR 'preanaesthetic medication' OR 'preanaesthetic treatment' OR 'preanesthetic medication' OR 'preanesthetic treatment' OR 'premedication' OR benzodiazepines) AND 'extubation'/exp
- 'thoracic surgery' AND 'preemptive analgesia'/exp AND 'postoperative pain'/exp
- 'thoracic surgery' AND 'halogenated anaesthetics' AND 'propofol'/exp AND ('inflammation'/exp OR 'acute inflammation' OR 'bacterial inflammation' OR 'inflammation' OR 'inflammation reaction' OR 'inflammation response' OR 'inflammatory condition' OR 'inflammatory lesion' OR 'inflammatory process' OR 'inflammatory reaction' OR 'inflammatory response' OR 'inflammatory syndrome' OR 'reaction, inflammation' OR 'response, inflammatory' OR 'serositis' OR 'sterile inflammation' OR 'systemic inflammation'/exp OR 'alveolar inflammation' OR 'pulmonary complications')
- 'thoracic surgery' AND 'opioid analgesics' AND ('pulmonary vasoconstriction' OR 'extubation time'/exp)^[[7]](#footnote-7)^
- 'thoracic surgery' AND ('neuromuscular blocking'/exp OR sugammaedx) AND ('postoperative complication'/exp OR 'complication, postoperative' OR 'complication, surgical' OR 'post-operative complication' OR 'post-operative complications' OR 'postoperative complication' OR 'postoperative complications' OR 'postsurgical complication' OR 'surgical complication')
- 'thoracic surgery' AND ('neuromuscular blocking'/exp OR sugammaedx)

***Locoregional analgesia***

*Pubmed:*

- (((thoracic surgery)) AND ((nerve blockade) OR (nerve block) AND (Epidural Anesthesia)) AND ((complication, intraoperative) OR (complication, postoperative)))
- (((thoracic surgery)) AND ((nerve blockade) OR (nerve block) AND (Epidural Anesthesia)) AND ((analgesics) OR (analgesic consumption)))
- (((thoracic surgery) AND (minimally invasive surgical procedures) OR (open surgery)) AND ((nerve blockade) OR (nerve block) AND (Epidural Anesthesia)) AND ((analgesics) OR (analgesic consumption) OR (postoperative pain)))
- (((thoracic surgery) AND (serratus anterior plane block)) AND ((epidural anesthesia)) AND ((analgesics) OR (analgesic consumption) OR (postoperative pain)))
- (((thoracic surgery) AND (dexamethasone) AND ((nerve blockade) OR (nerve block) AND (Epidural Anesthesia)) AND ((analgesics) OR (analgesic consumption) OR (postoperative pain)))
- (((lung resection) AND ((nerve blockade) OR (nerve block) AND (Epidural Anesthesia)) AND ((analgesics) OR (analgesic consumption) OR (postoperative pain)))
- (((lung resection) AND ((nerve blockade) OR (nerve block) OR (interfascial block) AND (Epidural Anesthesia)) AND ((analgesics) OR (analgesic consumption) OR (postoperative pain)))
- (((lung resection) AND ((anesthesia, general) AND (nerve blockade) OR (nerve block) AND (Epidural Anesthesia)) AND ((postoperative complications) OR (complication, postoperative) OR (hospitalization)))
- (((lung resection) AND ((erector spinae plane) OR (serratus plane))

*Embase:*

- 'thorax surgery'/exp AND ('paravertebral blockade'/exp OR 'paravertebral blockade') AND ('epidural anesthesia'/exp OR 'anaesthesia, epidural' OR 'anaesthesia, peridural' OR 'anesthesia, epidural' OR 'anesthesia, peridural' OR 'dural blocking' OR 'epidural anaesthesia' OR 'epidural anaesthetic agent' OR 'epidural anesthesia' OR 'epidural anesthetic agent' OR 'epidural block' OR 'epidural blockade' OR 'extradural anaesthesia' OR 'extradural anesthesia' OR 'extradural block' OR 'lumbar epidural anaesthesia' OR 'lumbar epidural anesthesia' OR 'lumbar peridural anaesthesia' OR 'lumbar peridural anesthesia' OR 'lumbar peridural block' OR 'peridural anaesthesia' OR 'peridural anesthesia' OR 'peridural block' OR 'peridural blocking') AND ('peroperative complication'/exp OR 'postoperative complication'/exp)
- 'thorax surgery'/exp AND ('paravertebral blockade'/exp OR 'paravertebral blockade') AND ('epidural anesthesia'/exp OR 'anaesthesia, epidural' OR 'anaesthesia, peridural' OR 'anesthesia, epidural' OR 'anesthesia, peridural' OR 'dural blocking' OR 'epidural anaesthesia' OR 'epidural anaesthetic agent' OR 'epidural anesthesia' OR 'epidural anesthetic agent' OR 'epidural block' OR 'epidural blockade' OR 'extradural anaesthesia' OR 'extradural anesthesia' OR 'extradural block' OR 'lumbar epidural anaesthesia' OR 'lumbar epidural anesthesia' OR 'lumbar peridural anaesthesia' OR 'lumbar peridural anesthesia' OR 'lumbar peridural block' OR 'peridural anaesthesia' OR 'peridural anesthesia' OR 'peridural block' OR 'peridural blocking') AND ('postoperative pain'/exp OR 'analgesic consumption' OR analgesics)
- 'thorax surgery'/exp AND 'minimally invasive procedure'/exp AND ('open surgery'/exp OR 'open surgery') AND ('postoperative pain'/exp OR 'analgesic consumption' OR analgesics)
- 'thorax surgery'/exp AND 'paravertebral blockade'/exp AND 'epidural blockade' AND ('peroperative complication'/exp OR 'complication, peroperative' OR 'intra-operative complication' OR 'intra-operative complications' OR 'intraoperative complication' OR 'intraoperative complications' OR 'per-operative complication' OR 'per-operative complications' OR 'peri-operative complication' OR 'peri-operative complications' OR 'perioperative complication' OR 'perioperative complications' OR 'peroperative complication' OR 'peroperative complications' OR 'postoperative complication'/exp)
- 'thorax surgery'/exp AND 'serratus anterior plane block'/exp AND 'epidural blockade' AND ('postoperative pain'/exp OR 'pain, postoperative' OR 'post operation pain' OR 'postoperative pain' OR 'analgesic agent'/exp OR 'agent, analgesic' OR 'analgesic' OR 'analgesic agent' OR 'analgesic drug' OR 'analgesic evaluation' OR 'analgesic tv 393' OR 'analgesics' OR 'analgesics, non narcotic' OR 'analgesics, non-narcotic' OR 'analgesics, topical' OR 'analgetic' OR 'analgetic agent' OR 'analgetic drug' OR 'anodyne' OR 'antalgic agent' OR 'non narcotic analgesic' OR 'non narcotic analgesic agent' OR 'nonnarcotic analgesic' OR 'nonnarcotic analgesic agent' OR 'analgesic use')^[[8]](#footnote-8)^
- 'thorax surgery'/exp AND 'dexamethasone'/exp AND 'neuromuscular blockade'
- ('lung resection'/exp OR 'lung resection' OR 'lung volume reduction surgery' OR 'lung, resected' OR 'pneumectomy' OR 'pneumonectomy' OR 'pneumonic resection' OR 'pneumoresection' OR 'pulmonary resection' OR 'pulmonectomy' OR 'resected lung' OR 'resection, lung' OR 'surgery, lung volume reduction') AND 'epidural anesthesia'/exp AND 'paravertebral nerve block'/exp AND ('postoperative pain'/exp OR 'pain, postoperative' OR 'post operation pain' OR 'postoperative pain' OR 'postoperative complication'/exp)
- ('lung resection'/exp OR 'lung resection' OR 'lung volume reduction surgery' OR 'lung, resected' OR 'pneumectomy' OR 'pneumonectomy' OR 'pneumonic resection' OR 'pneumoresection' OR 'pulmonary resection' OR 'pulmonectomy' OR 'resected lung' OR 'resection, lung' OR 'surgery, lung volume reduction') AND 'locoregional anesthesia'/exp AND 'general anesthesia'/exp AND ('postoperative complication'/exp OR 'hospitalization'/exp)^[[9]](#footnote-9)^
- ('lung resection'/exp OR 'lung resection' OR 'lung volume reduction surgery' OR 'lung, resected' OR 'pneumectomy' OR 'pneumonectomy' OR 'pneumonic resection' OR 'pneumoresection' OR 'pulmonary resection' OR 'pulmonectomy' OR 'resected lung' OR 'resection, lung' OR 'surgery, lung volume reduction') AND ('erector spinae plane block'/exp OR 'serratus plane block'/exp)

*Monitoring systems*

*Pubmed:*

- (((thoracic surgery) AND (lateral decubitus)) AND (anesthesia) AND ((blood pressure) OR (blood pressure monitoring)))
- (((one-lung ventilation) OR (single-lung ventilation)) AND (positioning))
- (((Pneumonectomy) OR (Lung Volume Reduction) OR (Partial Pneumonectomy)) AND ((electroencephalography) OR (EEG) OR (Electroencephalogram)))
- (((Pneumonectomy) OR (Lung Volume Reduction) OR (Partial Pneumonectomy)) AND ((consiousness monitors) OR (monitoring, intraoperative)))
- (((Pneumonectomy) OR (Lung Volume Reduction) OR (Partial Pneumonectomy)) AND ((pleural spaces) AND (chest drain)))
- (((Pneumonectomy) OR (Lung Volume Reduction) OR (Partial Pneumonectomy)) AND ((chest drain) AND (suction))
- ((thoracic surgery) AND ((intraoperative monitoring) OR (monitoring, intraoperative)) AND (blood pressure measurement))
- ((thoracic surgery) AND ((intraoperative monitoring) OR (monitoring, intraoperative)) AND (central venous catheterization) AND (hemodynamics)))
- ((thoracic surgery) AND ((intraoperative monitoring) OR (monitoring, intraoperative)) AND (bladder catheterization)))
- ((thoracic surgery) AND (monitoring urine output))

*Embase:*

- 'lateral decubitus' AND ('thorax surgery'/exp OR 'cardiothoracic surgery' OR 'chest surgery' OR 'chest wall surgery' OR 'surgery, chest' OR 'surgery, thoracic' OR 'surgery, thorax' OR 'thoracic operation' OR 'thoracic surgery' OR 'thoracic surgical procedures' OR 'thorax surgery') AND ('blood pressure monitoring'/exp OR '24 hour blood pressure' OR '24-hour blood pressure' OR 'ambulatory blood pressure monitoring' OR 'blood pressure monitoring' OR 'blood pressure monitoring, ambulatory' OR 'monitoring, ambulatory blood pressure' OR 'monitoring, blood pressure') OR 'perioperative monitoring'/exp OR 'monitoring, peroperative' OR 'perioperative monitoring' OR 'perisurgical monitoring' OR 'peroperative monitoring')
- ('one lung ventilation'/exp OR 'one lung ventilation' OR 'one-lung ventilation') AND 'tube positioning'
- ('one lung ventilation'/exp OR 'one lung ventilation' OR 'one-lung ventilation') AND 'tube placement'
- ('lung lobectomy'/exp OR 'lobectomy, lung' OR 'lung lobe resection' OR 'lung lobectomy' OR 'pneumolobectomy' OR 'pulmonary lobectomy') AND 'entropy'/exp
- ('lung lobectomy'/exp OR 'lobectomy, lung' OR 'lung lobe resection' OR 'lung lobectomy' OR 'pneumolobectomy' OR 'pulmonary lobectomy') AND ('perioperative monitoring'/exp OR 'monitoring, peroperative' OR 'perioperative monitoring' OR 'perisurgical monitoring' OR 'peroperative monitoring' OR 'electroencephalography'/exp OR 'electric encephalography' OR 'electrical encephalography' OR 'electro encephalography' OR 'electroencephalography')
- ('lung lobectomy'/exp OR 'lobectomy, lung' OR 'lung lobe resection' OR 'lung lobectomy' OR 'pneumolobectomy' OR 'pulmonary lobectomy') AND ('perioperative monitoring'/exp OR 'monitoring, peroperative' OR 'perioperative monitoring' OR 'perisurgical monitoring' OR 'peroperative monitoring')
- ('lung lobectomy'/exp OR 'lobectomy, lung' OR 'lung lobe resection' OR 'lung lobectomy' OR 'pneumolobectomy' OR 'pulmonary lobectomy') AND ('chest tube'/exp OR 'chest drain' OR 'suction'/exp) AND ('pleural spaces' OR 'air leaks' OR 'walking'/exp OR 'walking')
- 'thorax surgery'/exp AND ('perioperative monitoring'/exp OR 'monitoring, peroperative' OR 'perioperative monitoring' OR 'perisurgical monitoring' OR 'peroperative monitoring' OR 'central venous catheterization'/exp OR 'catheterisation, central venous' OR 'catheterization, central venous' OR 'central vein catheterisation' OR 'central vein catheterization' OR 'central venous catheterisation' OR 'central venous catheterization') AND ('blood pressure'/exp OR 'heart output'/exp OR 'electroencephalogram'/exp OR 'eeg' OR 'brain activity' OR 'brain electric activity' OR 'brain electrical activity' OR 'brain wave' OR 'brain waves' OR 'brainwave' OR 'brainwaves' OR 'e.e.g.' OR 'eeg activity' OR 'eeg analysis' OR 'electric encephalogram' OR 'electrical encephalogram' OR 'electro encephalogram' OR 'electroencephalogram' OR 'isoelectric eeg' OR 'body temperature'/exp OR 'body heat' OR 'body temperature' OR 'normothermia' OR 'temperature, body' OR 'urine output')
- 'thorax surgery'/exp AND ('perioperative monitoring'/exp OR 'monitoring, peroperative' OR 'perioperative monitoring' OR 'perisurgical monitoring' OR 'peroperative monitoring') AND 'cardiac output'
- 'thorax surgery'/exp AND ('perioperative monitoring'/exp OR 'monitoring, peroperative' OR 'perioperative monitoring' OR 'perisurgical monitoring' OR 'peroperative monitoring') AND 'body temperature'/exp
- 'thorax surgery'/exp AND ('perioperative monitoring'/exp OR 'monitoring, peroperative' OR 'perioperative monitoring' OR 'perisurgical monitoring' OR 'peroperative monitoring') AND ('urine volume'/exp OR 'urinary output' OR 'urinary volume' OR 'urine output' OR 'urine volume')
- 'thorax surgery'/exp AND ('perioperative monitoring'/exp OR 'monitoring, peroperative' OR 'perioperative monitoring' OR 'perisurgical monitoring' OR 'peroperative monitoring') AND ('electroencephalogram'/exp OR 'eeg' OR 'brain activity' OR 'brain electric activity' OR 'brain electrical activity' OR 'brain wave' OR 'brain waves' OR 'brainwave' OR 'brainwaves' OR 'e.e.g.' OR 'eeg activity' OR 'eeg analysis' OR 'electric encephalogram' OR 'electrical encephalogram' OR 'electro encephalogram' OR 'electroencephalogram' OR 'isoelectric eeg')
- 'thorax surgery'/exp AND 'neuromuscular blockade' AND ('perioperative monitoring'/exp OR 'monitoring, peroperative' OR 'perioperative monitoring' OR 'perisurgical monitoring' OR 'peroperative monitoring')^[[10]](#footnote-10)^
- 'thorax surgery'/exp AND 'neuromuscular blockade' AND 'monitoring'/exp

***Protective one-lung ventilation***

*Pubmed:*

- ((((pneumonectomy) OR (Lung Volume Reduction) OR (Partial Pneumonectomy) AND ((one-lung ventilation) OR (single-lung ventilation))) AND ((pulmonary ventilation) OR (ventilation) OR (protective ventilation)) AND ((postoperative complications) OR (complication, postoperative)))
- ((((pneumonectomy) OR (Lung Volume Reduction) OR (Partial Pneumonectomy) AND ((one-lung ventilation) OR (single-lung ventilation))) AND ((fluid therapy) OR (Oral Rehydration) OR (Oral Rehydration Therapy) OR ( Rehydration) OR (Rehydration Therapy, Oral) OR (Rehydration, Oral) OR (Therapy, Fluid) OR (Therapy, Oral Rehydration)) AND ((postoperative complications) OR (complication, postoperative)))

*Embase:*

- ('lung resection'/exp OR 'one lung ventilation'/exp OR 'one lung ventilation' OR 'one-lung ventilation') AND ('protective ventilation'/exp OR 'fluid restriction'/exp OR 'artificial ventilation'/exp) AND 'pulmonary complication'
- ('lung resection'/exp OR 'one lung ventilation'/exp OR 'one lung ventilation' OR 'one-lung ventilation') AND ('fluid therapy'/exp OR 'fluid therapy' OR 'parenteral fluid therapy' OR 'therapy, fluid') AND 'postoperative complication'/exp
- ('lung resection'/exp OR 'one lung ventilation'/exp OR 'one lung ventilation' OR 'one-lung ventilation') AND ('fluid therapy'/exp OR 'fluid therapy' OR 'parenteral fluid therapy' OR 'therapy, fluid') AND 'postoperative complication'/exp
- ('lung resection'/exp OR 'one lung ventilation'/exp OR 'one lung ventilation' OR 'one-lung ventilation') AND 'artificial ventilation'/exp AND 'pulmonary complications'

***Nasogastric feeding tube***

*Pubmed:*

- (((thoracic surgery) AND ((urinary catheters) AND (urinary tract infections) OR (urinary retention)))
- (((thoracic surgery) AND ((urinary catheters) AND ((anesthesia, epidural) OR (epidural anesthesia)) AND (urinary tract infections) OR (urinary retention)))
- (((thoracic surgery) AND ((intubation, nasogastric) OR (intubation, gastrointestinal) OR (nasogastric tube)) AND ((postoperative nausea and vomiting) OR (Emesis, Postoperative) OR (Nausea and Vomiting, Postoperative) OR (Nausea, Postoperative) OR (PONV) OR (Postoperative Emesis) OR (Postoperative Nausea) OR (Postoperative Vomiting) OR (Vomiting, Postoperative)))
- (((thoracic surgery) OR (pneumonectomy) OR (lung resection)) AND ((intubation, nasogastric) OR (intubation, gastrointestinal) OR (nasogastric tube)) AND ((postoperative nausea and vomiting) OR (Emesis, Postoperative) OR (Nausea and Vomiting, Postoperative) OR (Nausea, Postoperative) OR (PONV) OR (Postoperative Emesis) OR (Postoperative Nausea) OR (Postoperative Vomiting) OR (Vomiting, Postoperative)))
- (((thoracic surgery) OR (pneumonectomy) OR (lung resection)) AND ((intubation, nasogastric) OR (intubation, gastrointestinal) OR (nasogastric tube)) AND ((postoperative complications) OR (complication, Postoperative)))
- (((thoracic surgery) OR (pneumonectomy) OR (lung resection)) AND ((intubation, nasogastric) OR (intubation, gastrointestinal) OR (nasogastric tube)) AND ((enteral nutrition) OR (nutrition, enteral) OR (enteral feeding)))
- (((thoracic surgery) OR (pneumonectomy) OR (lung resection)) AND ((intubation, nasogastric) OR (intubation, gastrointestinal) OR (nasogastric tube)) AND ((oral nutrition)))^[[11]](#footnote-11)^
- (((thoracic surgery) OR (pneumonectomy) OR (lung resection)) AND ((intubation, nasogastric) OR (intubation, gastrointestinal) OR (nasogastric tube)) AND ((mobilization)))^‡‡‡^
- (((thoracic surgery) OR (pneumonectomy) OR (lung resection)) AND ((intubation, nasogastric) OR (intubation, gastrointestinal) OR (nasogastric tube)) AND ((recovery)))

*Embase:*

- 'thorax surgery'/exp AND 'urinary catheter'/exp AND ('urinary infections' OR 'urine retention'/exp OR 'ischuria' OR 'retention, urine' OR 'urinary retention' OR 'urine retention')
- ('thorax surgery'/exp OR 'epidural catheter'/exp OR 'flextip plus' OR 'flexitip plus' OR 'perifix fx' OR 'epidural catheter' OR 'epidural catheter, device' OR 'epidural catheter, device (physical object)' OR 'extradural catheter' OR 'epidural anesthesia'/exp) AND 'urinary catheter'/exp AND ('urinary infections' OR 'urine retention'/exp OR 'ischuria' OR 'retention, urine' OR 'urinary retention' OR 'urine retention')
- ('thorax surgery'/exp OR pneumonectomy OR 'lung resection'/exp) AND ('nasogastric tube'/exp OR 'flexiflo' OR 'ng (intubation)' OR 'ng tube' OR 'ngt (intubation)' OR 'nasoenteral tube' OR 'nasogastric feeding tube' OR 'nasogastric tube' OR 'nasogastric tube each' OR 'nasogastric tube misc. each' OR 'tube, nasogastric') AND ('postoperative nausea and vomiting'/exp OR 'ponv' OR 'postoperative nausea and vomiting')
- ('thorax surgery'/exp OR pneumonectomy OR 'lung resection'/exp) AND ('nasogastric tube'/exp OR 'flexiflo' OR 'ng (intubation)' OR 'ng tube' OR 'ngt (intubation)' OR 'nasoenteral tube' OR 'nasogastric feeding tube' OR 'nasogastric tube' OR 'nasogastric tube each' OR 'nasogastric tube misc. each' OR 'tube, nasogastric') AND 'postoperative complication'/exp
- ('thorax surgery'/exp OR pneumonectomy OR 'lung resection'/exp) AND ('nasogastric tube'/exp OR 'flexiflo' OR 'ng (intubation)' OR 'ng tube' OR 'ngt (intubation)' OR 'nasoenteral tube' OR 'nasogastric feeding tube' OR 'nasogastric tube' OR 'nasogastric tube each' OR 'nasogastric tube misc. each' OR 'tube, nasogastric') AND 'oral nutrition'
- ('thorax surgery'/exp OR pneumonectomy OR 'lung resection'/exp) AND ('nasogastric tube'/exp OR 'flexiflo' OR 'ng (intubation)' OR 'ng tube' OR 'ngt (intubation)' OR 'nasoenteral tube' OR 'nasogastric feeding tube' OR 'nasogastric tube' OR 'nasogastric tube each' OR 'nasogastric tube misc. each' OR 'tube, nasogastric') AND ('oral nutrition' OR 'enteric feeding'/exp)
- ('thorax surgery'/exp OR pneumonectomy OR 'lung resection'/exp) AND ('nasogastric tube'/exp OR 'flexiflo' OR 'ng (intubation)' OR 'ng tube' OR 'ngt (intubation)' OR 'nasoenteral tube' OR 'nasogastric feeding tube' OR 'nasogastric tube' OR 'nasogastric tube each' OR 'nasogastric tube misc. each' OR 'tube, nasogastric') AND ('mobilization'/exp OR 'ambulation' OR 'early ambulation' OR 'early mobilisation' OR 'early mobilization' OR 'mobilisation' OR 'mobilization' OR 'postoperative recovery'/exp)

***Fluid therapy***

*Pubmed:*

- (((thoracic surgery) AND ((fluid therapy) OR (water-electrolyte balance) OR (fluid balance)) AND ((postoperative complications) OR (complication, postoperative) AND (mortality)))
- (((thoracic surgery) AND ((fluid therapy) OR (water-electrolyte balance) OR (fluid balance)) AND ((normal saline)))
- (((thoracic surgery) AND ((fluid therapy) OR (water-electrolyte balance) OR (fluid balance)) AND ((normal saline) OR (sodium chloride) OR (hydroxyethyl starch)))
- (((thoracic surgery) AND ((fluid therapy) OR (water-electrolyte balance) OR (fluid balance)) AND ((hemoglobin concentration) AND (fluid volume)))
- (((thoracic surgery) AND ((hemoglobin concentration) AND (fluid volume)))
- (((thoracic surgery) OR (pneumonectomy) OR (lung resection)) AND ((hemoglobin concentration) AND (fluid volume)))
- (((thoracic surgery) OR (pneumonectomy) OR (lung resection)) AND ((hemoglobin) AND (fluid volume)))

*Embase:*

- ('thorax surgery'/exp OR 'lung resection'/exp OR 'lung resection' OR 'lung volume reduction surgery' OR 'lung, resected' OR 'pneumectomy' OR 'pneumonectomy' OR 'pneumonic resection' OR 'pneumoresection' OR 'pulmonary resection' OR 'pulmonectomy' OR 'resected lung' OR 'resection, lung' OR 'surgery, lung volume reduction') AND ('fluid therapy'/exp OR 'fluid therapy' OR 'parenteral fluid therapy' OR 'therapy, fluid') AND ('postoperative complication'/exp OR 'complication, postoperative' OR 'complication, surgical' OR 'post-operative complication' OR 'post-operative complications' OR 'postoperative complication' OR 'postoperative complications' OR 'postsurgical complication' OR 'surgical complication') AND 'mortality'/exp
- ('thorax surgery'/exp OR 'lung resection'/exp OR 'lung resection' OR 'lung volume reduction surgery' OR 'lung, resected' OR 'pneumectomy' OR 'pneumonectomy' OR 'pneumonic resection' OR 'pneumoresection' OR 'pulmonary resection' OR 'pulmonectomy' OR 'resected lung' OR 'resection, lung' OR 'surgery, lung volume reduction') AND 'sodium chloride'/exp AND 'postoperative complication'/exp
- ('thorax surgery'/exp OR 'lung resection'/exp OR 'lung resection' OR 'lung volume reduction surgery' OR 'lung, resected' OR 'pneumectomy' OR 'pneumonectomy' OR 'pneumonic resection' OR 'pneumoresection' OR 'pulmonary resection' OR 'pulmonectomy' OR 'resected lung' OR 'resection, lung' OR 'surgery, lung volume reduction') AND 'hydroxyethyl starch'
- ('thorax surgery'/exp OR 'lung resection'/exp OR 'lung resection' OR 'lung volume reduction surgery' OR 'lung, resected' OR 'pneumectomy' OR 'pneumonectomy' OR 'pneumonic resection' OR 'pneumoresection' OR 'pulmonary resection' OR 'pulmonectomy' OR 'resected lung' OR 'resection, lung' OR 'surgery, lung volume reduction') AND 'hemoglobin blood level'/exp AND ('monitoring'/exp OR 'monitoring')
- ('thorax surgery'/exp OR 'lung resection'/exp OR 'lung resection' OR 'lung volume reduction surgery' OR 'lung, resected' OR 'pneumectomy' OR 'pneumonectomy' OR 'pneumonic resection' OR 'pneumoresection' OR 'pulmonary resection' OR 'pulmonectomy' OR 'resected lung' OR 'resection, lung' OR 'surgery, lung volume reduction') AND 'goal-directed fluid therapy'
- ('thorax surgery'/exp OR 'lung resection'/exp OR 'lung resection' OR 'lung volume reduction surgery' OR 'lung, resected' OR 'pneumectomy' OR 'pneumonectomy' OR 'pneumonic resection' OR 'pneumoresection' OR 'pulmonary resection' OR 'pulmonectomy' OR 'resected lung' OR 'resection, lung' OR 'surgery, lung volume reduction') AND 'hemoglobin blood level'/exp AND ('fluid balance'/exp OR 'balance, fluid' OR 'balance, water' OR 'fluid balance' OR 'water balance' OR 'water equilibrium')

***PONV prevention***

*Pubmed:*

- (((lung resection) OR (pneumonectomy)) AND ((Postoperative Nausea and Vomiting) OR (Emesis, Postoperative) OR (Nausea and Vomiting, Postoperative) OR (Nausea, Postoperative) OR (PONV) OR (Postoperative Emesis) OR (Postoperative Nausea) OR (Postoperative Vomiting) OR (Vomiting, Postoperative)))
- (((lung resection) OR (pneumonectomy) OR (Lung Volume Reduction) OR (Partial Pneumonectomy)) AND Vomiting)
- (((lung resection) OR (pneumonectomy) OR (Lung Volume Reduction) OR (Partial Pneumonectomy)) AND (practice guideline))
- (((lung resection) OR (pneumonectomy) OR (Lung Volume Reduction) OR (Partial Pneumonectomy)) AND (chest tubes))
- (((lung resection) OR (pneumonectomy) OR (Lung Volume Reduction) OR (Partial Pneumonectomy)) AND (opioids) AND (postoperative nausea and vomiting)))
- (((lung resection) OR (pneumonectomy) OR (Lung Volume Reduction) OR (Partial Pneumonectomy)) AND (opioids) AND ((postoperative nausea and vomiting) OR (Emesis, Postoperative) OR (Nausea and Vomiting, Postoperative) OR (Nausea, Postoperative) OR (PONV) OR (Postoperative Emesis) OR (Postoperative Nausea) OR (Postoperative Vomiting) OR (Vomiting, Postoperative)))
- (((lung resection) OR (pneumonectomy) OR (Lung Volume Reduction) OR (Partial Pneumonectomy)) AND ((inhaled anesthesia) OR (intravenous anesthesia)) AND ((postoperative nausea and vomiting) OR (Emesis, Postoperative) OR (Nausea and Vomiting, Postoperative) OR (Nausea, Postoperative) OR (PONV) OR (Postoperative Emesis) OR (Postoperative Nausea) OR (Postoperative Vomiting) OR (Vomiting, Postoperative)))
- (((lung resection) OR (pneumonectomy) OR (Lung Volume Reduction) OR (Partial Pneumonectomy)) AND ((anesthesia, regional) OR (anesthesia, general)) AND ((postoperative nausea and vomiting) OR (Emesis, Postoperative) OR (Nausea and Vomiting, Postoperative) OR (Nausea, Postoperative) OR (PONV) OR (Postoperative Emesis) OR (Postoperative Nausea) OR (Postoperative Vomiting) OR (Vomiting, Postoperative)))
- (((lung resection) OR (pneumonectomy) OR (Lung Volume Reduction) OR (Partial Pneumonectomy)) AND ((antiemetics)) AND ((postoperative nausea and vomiting) OR (Emesis, Postoperative) OR (Nausea and Vomiting, Postoperative) OR (Nausea, Postoperative) OR (PONV) OR (Postoperative Emesis) OR (Postoperative Nausea) OR (Postoperative Vomiting) OR (Vomiting, Postoperative)))
- (((lung resection) OR (pneumonectomy) OR (Lung Volume Reduction) OR (Partial Pneumonectomy)) AND ((antiemetics) OR (prophylaxis)) AND ((postoperative nausea and vomiting) OR (Emesis, Postoperative) OR (Nausea and Vomiting, Postoperative) OR (Nausea, Postoperative) OR (PONV) OR (Postoperative Emesis) OR (Postoperative Nausea) OR (Postoperative Vomiting) OR (Vomiting, Postoperative)))
- (((lung resection) OR (pneumonectomy) OR (Lung Volume Reduction) OR (Partial Pneumonectomy)) AND ((glucocorticoids) OR (prophylaxis)) AND ((postoperative nausea and vomiting) OR (Emesis, Postoperative) OR (Nausea and Vomiting, Postoperative) OR (Nausea, Postoperative) OR (PONV) OR (Postoperative Emesis) OR (Postoperative Nausea) OR (Postoperative Vomiting) OR (Vomiting, Postoperative)))
- (((lung resection) OR (pneumonectomy) OR (Lung Volume Reduction) OR (Partial Pneumonectomy)) AND ((serotonin antagonists) OR (5-HT antagonists) OR (prophylaxis)) AND ((postoperative nausea and vomiting) OR (Emesis, Postoperative) OR (Nausea and Vomiting, Postoperative) OR (Nausea, Postoperative) OR (PONV) OR (Postoperative Emesis) OR (Postoperative Nausea) OR (Postoperative Vomiting) OR (Vomiting, Postoperative)))
- (((lung resection) OR (pneumonectomy) OR (Lung Volume Reduction) OR (Partial Pneumonectomy)) AND (fluid therapy) AND ((postoperative nausea and vomiting) OR (Emesis, Postoperative) OR (Nausea and Vomiting, Postoperative) OR (Nausea, Postoperative) OR (PONV) OR (Postoperative Emesis) OR (Postoperative Nausea) OR (Postoperative Vomiting) OR (Vomiting, Postoperative)))

*Embase:*

- ('lung resection' OR 'lung volume reduction surgery' OR 'lung, resected' OR 'pneumectomy' OR 'pneumonectomy' OR 'pneumonic resection' OR 'pneumoresection' OR 'pulmonary resection' OR 'pulmonectomy' OR 'resected lung' OR 'resection, lung' OR 'surgery, lung volume reduction' OR 'lung resection'/exp) AND 'practice guideline'/exp AND ('postoperative nausea and vomiting'/exp OR 'ponv' OR 'postoperative nausea and vomiting')
- ('lung resection' OR 'lung volume reduction surgery' OR 'lung, resected' OR 'pneumectomy' OR 'pneumonectomy' OR 'pneumonic resection' OR 'pneumoresection' OR 'pulmonary resection' OR 'pulmonectomy' OR 'resected lung' OR 'resection, lung' OR 'surgery, lung volume reduction' OR 'lung resection'/exp) AND ('postoperative nausea and vomiting'/exp OR 'ponv' OR 'postoperative nausea and vomiting')
- ('lung resection' OR 'lung volume reduction surgery' OR 'lung, resected' OR 'pneumectomy' OR 'pneumonectomy' OR 'pneumonic resection' OR 'pneumoresection' OR 'pulmonary resection' OR 'pulmonectomy' OR 'resected lung' OR 'resection, lung' OR 'surgery, lung volume reduction' OR 'lung resection'/exp) AND 'chest tube'/exp AND ('postoperative pain'/exp OR 'pain, postoperative' OR 'post operation pain' OR 'postoperative pain' OR 'air leak'/exp)
- ('lung resection' OR 'lung volume reduction surgery' OR 'lung, resected' OR 'pneumectomy' OR 'pneumonectomy' OR 'pneumonic resection' OR 'pneumoresection' OR 'pulmonary resection' OR 'pulmonectomy' OR 'resected lung' OR 'resection, lung' OR 'surgery, lung volume reduction' OR 'lung resection'/exp) AND ('intravenous anesthesia'/exp OR 'inhalation anesthetic agent'/exp) AND 'postoperative nausea and vomiting'/exp
- ('lung resection' OR 'lung volume reduction surgery' OR 'lung, resected' OR 'pneumectomy' OR 'pneumonectomy' OR 'pneumonic resection' OR 'pneumoresection' OR 'pulmonary resection' OR 'pulmonectomy' OR 'resected lung' OR 'resection, lung' OR 'surgery, lung volume reduction' OR 'lung resection'/exp) AND ('antiemetic agent'/exp OR 'anti emetic agent' OR 'antiemetic' OR 'antiemetic agent' OR 'antiemetic drug' OR 'antiemetics')
- ('lung resection' OR 'lung volume reduction surgery' OR 'lung, resected' OR 'pneumectomy' OR 'pneumonectomy' OR 'pneumonic resection' OR 'pneumoresection' OR 'pulmonary resection' OR 'pulmonectomy' OR 'resected lung' OR 'resection, lung' OR 'surgery, lung volume reduction' OR 'lung resection'/exp) AND 'prevention of postoperative nausea and vomiting'
- ('lung resection'/exp OR 'lung resection' OR 'lung volume reduction surgery' OR 'lung, resected' OR 'pneumectomy' OR 'pneumonectomy' OR 'pneumonic resection' OR 'pneumoresection' OR 'pulmonary resection' OR 'pulmonectomy' OR 'resected lung' OR 'resection, lung' OR 'surgery, lung volume reduction' OR pneumonectomy) AND ('glucocorticoid'/exp OR 'serotonin antagonist'/exp OR '5 ht antagonist' OR '5 ht blocker' OR '5 ht blocking agent' OR '5 ht receptor antagonist' OR '5 ht receptor blocker' OR '5 ht receptor blocking agent' OR '5 hydroxytryptamine antagonist' OR '5 hydroxytryptamine blocker' OR '5 hydroxytryptamine blocking agent' OR '5 hydroxytryptamine receptor antagonist' OR '5 hydroxytryptamine receptor blocker' OR '5 hydroxytryptamine receptor blocking agent' OR '5ht antagonist' OR '5ht blocker' OR '5ht blocking agent' OR '5ht receptor antagonist' OR '5ht receptor blocker' OR '5ht receptor blocking agent' OR 'anti 5 ht agent' OR 'anti 5 hydroxytryptamine agent' OR 'anti 5ht agent' OR 'antiserotonergic agent' OR 'antiserotonergic drug' OR 'antiserotonic' OR 'antiserotonin' OR 'antiserotonin action' OR 'antiserotonin agent' OR 'antiserotonin drug' OR 'antiserotoninergic agent' OR 'antiserotoninergic drug' OR 'serotonin antagonist' OR 'serotonin antagonists' OR 'serotonin blocker' OR 'serotonin blocking agent' OR 'serotonin receptor antagonist' OR 'serotonin receptor antagonists' OR 'serotonin receptor blocker' OR 'serotonin receptor blocking agent' OR 'antiemetic agent'/exp OR 'anti emetic agent' OR 'antiemetic' OR 'antiemetic agent' OR 'antiemetic drug' OR 'antiemetics') AND 'postoperative nausea and vomiting'/exp^[[12]](#footnote-12)^
- ('lung resection'/exp OR 'lung resection' OR 'lung volume reduction surgery' OR 'lung, resected' OR 'pneumectomy' OR 'pneumonectomy' OR 'pneumonic resection' OR 'pneumoresection' OR 'pulmonary resection' OR 'pulmonectomy' OR 'resected lung' OR 'resection, lung' OR 'surgery, lung volume reduction' OR pneumonectomy) AND ('glucocorticoid'/exp OR 'serotonin antagonist'/exp OR '5 ht antagonist' OR '5 ht blocker' OR '5 ht blocking agent' OR '5 ht receptor antagonist' OR '5 ht receptor blocker' OR '5 ht receptor blocking agent' OR '5 hydroxytryptamine antagonist' OR '5 hydroxytryptamine blocker' OR '5 hydroxytryptamine blocking agent' OR '5 hydroxytryptamine receptor antagonist' OR '5 hydroxytryptamine receptor blocker' OR '5 hydroxytryptamine receptor blocking agent' OR '5ht antagonist' OR '5ht blocker' OR '5ht blocking agent' OR '5ht receptor antagonist' OR '5ht receptor blocker' OR '5ht receptor blocking agent' OR 'anti 5 ht agent' OR 'anti 5 hydroxytryptamine agent' OR 'anti 5ht agent' OR 'antiserotonergic agent' OR 'antiserotonergic drug' OR 'antiserotonic' OR 'antiserotonin' OR 'antiserotonin action' OR 'antiserotonin agent' OR 'antiserotonin drug' OR 'antiserotoninergic agent' OR 'antiserotoninergic drug' OR 'serotonin antagonist' OR 'serotonin antagonists' OR 'serotonin blocker' OR 'serotonin blocking agent' OR 'serotonin receptor antagonist' OR 'serotonin receptor antagonists' OR 'serotonin receptor blocker' OR 'serotonin receptor blocking agent' OR 'antiemetic agent'/exp OR 'anti emetic agent' OR 'antiemetic' OR 'antiemetic agent' OR 'antiemetic drug' OR 'antiemetics') AND ('nausea and vomiting'/exp OR 'nausea and vomiting')
- ('lung resection'/exp OR 'lung resection' OR 'lung volume reduction surgery' OR 'lung, resected' OR 'pneumectomy' OR 'pneumonectomy' OR 'pneumonic resection' OR 'pneumoresection' OR 'pulmonary resection' OR 'pulmonectomy' OR 'resected lung' OR 'resection, lung' OR 'surgery, lung volume reduction' OR pneumonectomy) AND ('fluid therapy'/exp OR 'fluid therapy' OR 'parenteral fluid therapy' OR 'therapy, fluid') AND ('postoperative nausea and vomiting'/exp OR 'ponv' OR 'postoperative nausea and vomiting')

***Chest drain***

*Pubmed:*

- (((pneumonectomy) OR (Lung Volume Reduction) OR (Partial Pneumonectomy) OR (wedge resection)) AND (chest tubes))
- (((pneumonectomy) OR (Lung Volume Reduction) OR (Partial Pneumonectomy) OR (wedge resection)) AND (chest tubes) AND ((postoperative complications) OR (complication, postoperative) OR (pneumothorax) OR (arrhythmias, cardiac)))

*Embase:*

- ('lung lobectomy'/exp OR 'lobectomy, lung' OR 'lung lobe resection' OR 'lung lobectomy' OR 'pneumolobectomy' OR 'pulmonary lobectomy' OR 'wedge resection'/exp) AND ('chest tube'/exp OR 'pleur--evac' OR 'pleuraflow' OR 'chest drain' OR 'chest drain (physical object)' OR 'chest drainage system' OR 'chest tube' OR 'chest tube insertion kit' OR 'chest tubes' OR 'intercostal drain' OR 'tube, chest' OR 'tubes, chest') AND ('postoperative complication'/exp OR 'complication, postoperative' OR 'complication, surgical' OR 'post-operative complication' OR 'post-operative complications' OR 'postoperative complication' OR 'postoperative complications' OR 'postsurgical complication' OR 'surgical complication' OR arrhythmias OR 'pneumothorax'/exp OR 'bilateral pneumothorax' OR 'chronic pneumothorax' OR 'pneumothorax' OR 'pneumothorax, chronic' OR 'pneumothorax, valvular' OR 'valvular pneumothorax')

**Postoperative phase**

***Multimodal analgesia and imaging***

*Pubmed:*

- (((thoracic surgery) OR (Pneumonectomy) NOT (cardiac surgery)) AND ((analgesia) AND ((pain, postoperative) OR (postoperative pain)))
- (((thoracic surgery) OR (Pneumonectomy) NOT (cardiac surgery)) AND ((analgesia) AND ((complication, postoperative) OR (postoperative complications)))
- (((thoracic surgery) OR (Pneumonectomy) NOT (cardiac surgery)) AND ((Thoracic Surgery, Video-Assisted) OR (thoracotomy)) AND ((pain, postoperative) OR (postoperative pain)))
- (((Thoracic Surgery, Video-Assisted) OR (thoracotomy) NOT (cardiac surgery)) AND ((pain, postoperative) OR (postoperative pain)))
- (((Thoracic Surgery, Video-Assisted) OR (Surgery, Thoracic, Video-Assisted) OR (VATS) OR (Video-Assisted Thoracic Surgery) OR (Video-Assisted Thoracoscopic Surgery) NOT (cardiac surgery)) AND ((pain, postoperative) OR (postoperative pain)))
- (((Thoracic Surgery, Video-Assisted) OR (Surgery, Thoracic, Video-Assisted) OR (VATS) OR (Video-Assisted Thoracic Surgery) OR (Video-Assisted Thoracoscopic Surgery) NOT (cardiac surgery)) AND ((pain, postoperative) OR (postoperative pain) OR (postoperative complications) OR (complication, postoperative)))
- ((lung resection) OR (pneumonectomy) OR (Thoracic Surgery, Video-Assisted) OR (Surgery, Thoracic, Video-Assisted) OR (VATS) OR (Video-Assisted Thoracic Surgery) OR (Video-Assisted Thoracoscopic Surgery) NOT (cardiac surgery)) AND ((pain, postoperative) OR (postoperative pain)))
- ((lung resection) OR (pneumonectomy) NOT (cardiac surgery)) AND (chest tubes) AND ((pain, postoperative) OR (postoperative pain)))
- ((lung resection) OR (pneumonectomy) NOT (cardiac surgery)) AND (pain measurement))
- ((lung resection) OR (pneumonectomy) NOT (cardiac surgery)) AND ((pain measurement) OR (Analgesia Tests) OR (Analog Pain Scale) OR (Analogue Pain Scale) OR (Assessment, Pain) OR (Formalin Test) OR (McGill Pain Questionnaire) OR (McGill Pain Scale) OR (Nociception Tests) OR (Pain Assessment) OR (Tourniquet Pain Test) OR (Visual Analog Pain Scale) OR (Visual Analogue Pain Scale)))
- ((lung resection) OR (pneumonectomy) NOT (cardiac surgery)) AND ((pain measurement) OR (Analgesia Tests) OR (Analog Pain Scale) OR (Analogue Pain Scale) OR (Assessment, Pain) OR (Formalin Test) OR (McGill Pain Questionnaire) OR (McGill Pain Scale) OR (Nociception Tests) OR (Pain Assessment) OR (Tourniquet Pain Test) OR (Visual Analog Pain Scale) OR (Visual Analogue Pain Scale)))
- ((lung resection) OR (pneumonectomy) NOT (cardiac surgery)) AND (acute pain service))
- ((lung resection) OR (pneumonectomy) NOT (cardiac surgery)) AND ((acute pain service) OR (Multidisciplinary Pain Centers) OR (Multidisciplinary Pain Clinics) OR (Pain Centers) OR (Pain Relief Units) OR (Pain Service, Acute)))
- ((lung resection) OR (pneumonectomy) NOT (cardiac surgery)) AND ((postoperative pain) OR (pain, postoperative)))

*Embase:*

- 'thorax surgery'/exp NOT 'cardiac surgery' AND ('multimodal analgesia'/exp OR 'patient controlled analgesia'/exp OR 'analgesia, patient controlled' OR 'analgesia, patient-controlled' OR 'patient controlled anaesthesia' OR 'patient controlled analgesia' OR 'patient controlled anesthesia' OR 'self anaesthesia' OR 'self anesthesia') AND ('continuous infusion'/exp OR 'continuous drug infusion' OR 'continuous infusion') AND 'postoperative pain'/exp
- 'thorax surgery'/exp NOT 'cardiac surgery' AND ('multimodal analgesia'/exp OR 'patient controlled analgesia'/exp OR 'analgesia, patient controlled' OR 'analgesia, patient-controlled' OR 'patient controlled anaesthesia' OR 'patient controlled analgesia' OR 'patient controlled anesthesia' OR 'self anaesthesia' OR 'self anesthesia') AND ('continuous infusion'/exp OR 'continuous drug infusion' OR 'continuous infusion') AND ('postoperative complication'/exp OR 'complication, postoperative' OR 'complication, surgical' OR 'post-operative complication' OR 'post-operative complications' OR 'postoperative complication' OR 'postoperative complications' OR 'postsurgical complication' OR 'surgical complication')
- ('lung resection'/exp OR 'lung resection' OR 'lung volume reduction surgery' OR 'lung, resected' OR 'pneumectomy' OR 'pneumonectomy' OR 'pneumonic resection' OR 'pneumoresection' OR 'pulmonary resection' OR 'pulmonectomy' OR 'resected lung' OR 'resection, lung' OR 'surgery, lung volume reduction') AND ('risk factor'/exp OR 'relative risk' OR 'risk factor' OR 'risk factors') AND 'postoperative pain'/exp
- 'lung resection'/exp AND 'video assisted thoracoscopic surgery'/exp AND ('thoracotomy'/exp OR 'chest operation' OR 'incision, pleura' OR 'pleura incision' OR 'pleuracotomy' OR 'pleural incision' OR 'pleurotomy' OR 'rethoracotomy' OR 'thoracotomy') AND 'postoperative pain'/exp
- 'lung resection'/exp AND ('chest tube'/exp OR 'pleur--evac' OR 'pleuraflow' OR 'chest drain' OR 'chest drain (physical object)' OR 'chest drainage system' OR 'chest tube' OR 'chest tube insertion kit' OR 'chest tubes' OR 'intercostal drain' OR 'tube, chest' OR 'tubes, chest') AND 'postoperative pain'/exp
- 'lung resection'/exp AND 'pain assessment'/exp AND 'postoperative pain'/exp
- 'lung resection'/exp AND ('neuroaxial anesthesia'/exp OR 'local anesthetic agent'/exp) AND 'postoperative pain'/exp
- 'lung resection'/exp AND ('practice guideline'/exp OR 'clinical practice guidelines' OR 'guidelines' OR 'guidelines as topic' OR 'practice guideline' OR 'practice guidelines' OR 'practice guidelines as topic') AND 'postoperative pain'/exp
- 'lung resection'/exp AND ('magnesium sulfate'/exp OR 'alpha-2 agonist' OR 'ketamine'/exp OR 'corticosteroid'/exp) AND 'postoperative pain'/exp
- 'lung resection'/exp AND 'serratus plane block'/exp AND 'postoperative pain'/exp^[[13]](#footnote-13)^
- 'lung resection'/exp AND 'postoperative x-ray'
- 'lung resection'/exp AND 'thorax radiography'/exp AND 'early detection of complications'
- 'lung resection'/exp AND 'thorax radiography'/exp AND 'postoperative complication'/exp

***Nutrition***

*Pubmed:*

- (((lung resection) OR (pneumonectomy)) AND ((fasting) OR (feeding)))
- (((lung resection) OR (pneumonectomy)) AND ((postoperative nutrition)))

*Embase:*

- ('lung resection'/exp OR 'lung resection' OR 'lung volume reduction surgery' OR 'lung, resected' OR 'pneumectomy' OR 'pneumonectomy' OR 'pneumonic resection' OR 'pneumoresection' OR 'pulmonary resection' OR 'pulmonectomy' OR 'resected lung' OR 'resection, lung' OR 'surgery, lung volume reduction' OR 'major surgery'/exp OR 'major surgery') AND 'postoperative fasting'
- ('lung resection'/exp OR 'lung resection' OR 'lung volume reduction surgery' OR 'lung, resected' OR 'pneumectomy' OR 'pneumonectomy' OR 'pneumonic resection' OR 'pneumoresection' OR 'pulmonary resection' OR 'pulmonectomy' OR 'resected lung' OR 'resection, lung' OR 'surgery, lung volume reduction' OR 'major surgery'/exp OR 'major surgery') AND 'postoperative feeding’
- ('lung resection'/exp OR 'major surgery'/exp) AND fasting
- ('lung resection'/exp OR 'major surgery'/exp) AND (fasting OR 'eating'/exp)

***Mobilization***

*Pubmed:*

- (((lung resection) OR (pneumonectomy)) AND ((ambulation) OR (oral feeding)))
- (((lung resection) OR (pneumonectomy)) AND ((early ambulation) OR (early mobilization)

*Embase:*

- ('lung resection'/exp OR 'video assisted thoracoscopic surgery'/exp) AND ('early mobilization' OR 'early ambulation')
- ('lung resection'/exp OR 'video assisted thoracoscopic surgery'/exp) AND 'major resection' OR 'minor resection'

***Lung function***

*Pubmed:*

- (((Thoracic surgery) OR (lung resection) OR (pneumonectomy) NOT (cardiac surgery)) AND ((non invasive ventilation) OR (non-invasive ventilation) OR (mechanical ventilation) OR (ventilation, mechanical)))
- (((lung resection) OR (pneumonectomy) NOT (cardiac surgery)) AND ((non invasive ventilation) OR (non-invasive ventilation) OR (mechanical ventilation) OR (ventilation, mechanical)))
- ((thoracic surgery) OR (lung resection) OR (pneumonectomy) NOT (cardiac surgery)) AND ((non invasive ventilation) OR (non-invasive ventilation) OR (mechanical ventilation) OR (ventilation, mechanical)) AND (((respiratory function tests) OR (Function Test, Pulmonary) OR (Function Tests, Pulmonary) OR (Lung Function Tests) OR (Pulmonary Function Test) OR (Pulmonary Function Tests) OR (Test, Pulmonary Function) OR (Tests, Pulmonary Function)))
- (((thoracic surgery) OR (lung resection) OR (pneumonectomy) NOT (cardiac surgery)) AND ((non invasive ventilation) OR (non-invasive ventilation) OR (mechanical ventilation) OR (ventilation, mechanical)) AND (((postoperative complications) OR (complication, postoperative)))
- (((thoracic surgery) OR (lung resection) OR (pneumonectomy) NOT (cardiac surgery)) AND ((non invasive ventilation) OR (non-invasive ventilation) OR (mechanical ventilation) OR (ventilation, mechanical)) AND (mortality))
- (((thoracic surgery) OR (lung resection) OR (pneumonectomy) NOT (cardiac surgery)) AND ((non invasive ventilation) OR (non-invasive ventilation) OR (mechanical ventilation) OR (ventilation, mechanical)) AND ((respiratory failure) OR (respiratory depression) OR (respiratory insufficiency) OR (reintubation) OR (hospital-acquired pneumonia)))

*Embase:*

- ('thorax surgery'/exp OR 'lung resection'/exp) NOT 'heart surgery'/exp AND ('noninvasive ventilation'/exp OR 'non invasive ventilation' OR 'noninvasive ventilation') AND ('lung function'/exp OR 'function, lung' OR 'lung function' OR 'pulmonary function' OR 'regional lung function')
- ('thorax surgery'/exp OR 'lung resection'/exp) NOT 'heart surgery'/exp AND ('noninvasive ventilation'/exp OR 'non invasive ventilation' OR 'noninvasive ventilation') AND 'intensive care unit'/exp
- ('thorax surgery'/exp OR 'lung resection'/exp) NOT 'heart surgery'/exp AND ('noninvasive ventilation'/exp OR 'non invasive ventilation' OR 'noninvasive ventilation') AND ('postoperative complications' OR 'mortality'/exp OR 'hospital acquired pneumonia'/exp OR 'hospital acquired pneumonia' OR 'postoperative respiratory failure')
- ('thorax surgery'/exp OR 'lung resection'/exp) NOT 'heart surgery'/exp AND ('noninvasive ventilation'/exp OR 'non invasive ventilation' OR 'noninvasive ventilation') AND 'oxygen therapy'/exp AND ('postoperative complications' OR 'mortality'/exp OR 'hospital acquired pneumonia'/exp OR 'hospital acquired pneumonia' OR 'postoperative respiratory failure')
- ('thorax surgery'/exp OR 'lung resection'/exp) NOT 'heart surgery'/exp AND ('noninvasive ventilation'/exp OR 'non invasive ventilation' OR 'noninvasive ventilation' OR 'oxygen therapy'/exp) AND ('airway remodeling'/exp OR 'airway remodeling' OR 'airway remodelling' OR 'airway wall remodeling' OR 'airway wall remodelling')

***Postoperative arrhythmias***

*Pubmed:*

- (((lung resection) OR (pneumonectomy)) AND ((arrhythmias, cardiac) OR (Arrhythmia) OR (Cardiac Arrhythmia) OR (Cardiac Arrhythmias) OR (atrial fibrillation)))
- (((lung resection) OR (pneumonectomy)) AND ((arrhythmias, cardiac) OR (Arrhythmia) OR (Cardiac Arrhythmia) OR (Cardiac Arrhythmias) OR (atrial fibrillation)) AND ((postoperative complications) OR (complication, postoperative)))
- (((lung resection) OR (pneumonectomy)) AND ((atrial fibrillation)))

*Embase:*

- ('lung lobectomy'/exp OR 'lobectomy, lung' OR 'lung lobe resection' OR 'lung lobectomy' OR 'pneumolobectomy' OR 'pulmonary lobectomy') AND 'minimally invasive procedure'/exp AND ('thoracotomy'/exp OR 'chest operation' OR 'incision, pleura' OR 'pleura incision' OR 'pleuracotomy' OR 'pleural incision' OR 'pleurotomy' OR 'rethoracotomy' OR 'thoracotomy') AND ('heart arrhythmia'/exp OR 'atrial fibrillation'/exp)
- ('lung lobectomy'/exp OR 'lobectomy, lung' OR 'lung lobe resection' OR 'lung lobectomy' OR 'pneumolobectomy' OR 'pulmonary lobectomy') AND ('minimally invasive procedure'/exp OR 'thoracotomy'/exp) AND ('heart arrhythmia'/exp OR 'atrial fibrillation'/exp)
- ('lung lobectomy'/exp OR 'lobectomy, lung' OR 'lung lobe resection' OR 'lung lobectomy' OR 'pneumolobectomy' OR 'pulmonary lobectomy') AND ('practice guideline'/exp OR 'clinical practice guidelines' OR 'guidelines' OR 'guidelines as topic' OR 'practice guideline' OR 'practice guidelines' OR 'practice guidelines as topic') AND ('heart arrhythmia'/exp OR 'atrial fibrillation'/exp)

1. All searches filtered by English language, humans, and clinical trials/reviews, except where indicated by an asterisk, where no filters were applied. [↑](#footnote-ref-1)
2. Filters for age and study type applied to all Embase searches [↑](#footnote-ref-2)
3. No references found [↑](#footnote-ref-3)
4. Most references relate to cardiac surgery [↑](#footnote-ref-4)
5. No references found [↑](#footnote-ref-5)
6. No references found [↑](#footnote-ref-6)
7. No references found [↑](#footnote-ref-7)
8. No references found [↑](#footnote-ref-8)
9. No references found [↑](#footnote-ref-9)
10. No references found [↑](#footnote-ref-10)
11. No references found. [↑](#footnote-ref-11)
12. No references found [↑](#footnote-ref-12)
13. No references found [↑](#footnote-ref-13)
